# Quercetin exhibits multi-target anti-allergic effects in animal models: a systematic review and meta-analysis of preclinical studies

**DOI:** 10.3389/fphar.2025.1673712

**Published:** 2025-11-20

**Authors:** Zeyi Lv, Zhuo Pan, Yue Huang, Hao Yang, Xinrong Li

**Affiliations:** 1 Chengdu University of Traditional Chinese Medicine, Chengdu, China; 2 Hospital of Chengdu University of Traditional Chinese Medicine, Chengdu, China

**Keywords:** quercetin, allergic diseases, allergic rhinitis, meta-analysis, animal model, systematic review

## Abstract

**Background:**

Quercetin is a naturally occurring flavonoid widely present in fruits, vegetables and tea with multiple pharmacological activities, including immunomodulatory, anti-allergic, antioxidant and anti-inflammatory properties. Preclinical studies have indicated the potential to ameliorate allergic symptoms in animal models, but comprehensive synthesis is still scarce.

**Objective:**

This meta-analysis was conducted to summarize the therapeutic effects of quercetin in allergic disease models and explore its potential mechanisms.

**Methods:**

According to PRISMA recommendations, preclinical studies were extracted from PubMed, Web of Science and Embase databases. Thirteen eligible studies were extracted for quantitative synthesis analysis. In total, 13 studies using murine models (BALB/c, C57BL/6 mice, SKH-1 hairless mice and NC/Nga mice; Wistar and Sprague-Dawley rats) were included. The most closely related biomarkers were total IgE, OVA-specific IgE, histamine, inflammatory cytokines (IL-4, IL-5, IL-10, TNF-α, IFN-γ), and immune cell populations (macrophages, lymphocytes, eosinophils, neutrophils). Review Manager 5.4 software was used for analysis, and standardized mean differences (SMD) and 95% confidence intervals (CI) were calculated under a random-effects model.

**Results:**

The meta-analysis showed that quercetin significantly decreased the expression of total IgE, OVA-specific IgE, and histamine, and suppressed the infiltration of eosinophils, macrophages, and lymphocytes. Cytokine profiling showed that quercetin significantly suppressed the expression of IL-4 and TNF-α, and increased the expression of IFN-γ, which may contribute to the underlying anti-inflammatory mechanism of quercetin through Th1/Th2 immune rebalancing.

**Conclusion:**

Quercetin exhibits strong anti-allergic effects in preclinical models through suppression of IgE, modulation of immune cells, regulation of cytokine network, and reduction of histamine. However, large inter-study heterogeneity and methodological limitations in original studies should be cautiously interpreted. Application in clinical settings should be carefully evaluated through well-designed trials to validate safety, efficacy, and molecular mechanisms in human populations.

## Introduction

Allergic diseases (AD) are a heterogeneous group of hypersensitivity diseases caused by deviant immune responses against normally innocuous environmental antigens. Allergic diseases are a continuum of immune-mediated diseases in which the protective barriers of the body, consisting of both the innate and adaptive immune responses, recognize harmless environmental substances as foreign and induce exaggerated responses involving both immune cells and epithelial tissues. Typical examples are asthma, eczema (atopic dermatitis), hay fever (allergic rhinitis), eye allergies, chronic sinus inflammation, and food sensitivities now impact more than one-quarter of people in developed nations. Scarily, these are also on the rise in developing countries too, posing serious health threats and placing a strain on economies. The high worldwide incidence of these hypersensitivity diseases indicates their growing importance as a global public health problem.

Some of the causes like pollution, climate change, reduction in biodiversity, urbanization, change in lifestyle and dietary habits have been implicated for the important rise in allergic cases (Agache and Akdis, 2019; Aw Yong et al., 2020; Yao et al., 2021; Bousquet et al., 2020a). Scarily, these are also on the rise in developing countries too, posing serious health threats and placing a strain on economies (Ogulur et al., 2021). The high worldwide incidence of these hypersensitivity diseases indicates their growing importance as a global public health problem. Some of the causes like pollution, climate change, reduction in biodiversity, urbanization, change in lifestyle and dietary habits have been implicated for the important rise in allergic cases (Wang et al., 2023).

The World Health Organization (WHO) ranks allergic diseases among the top three conditions requiring prevention and management in the 21st century. Although broadly systemic in nature, these disorders typically present as “local diseases”, any of which can progress to life-threatening anaphylaxis in severe attacks.

The burdens of allergic diseases are tremendous, with extremely high prevalence rates.

It is estimated that 500 million people suffer from AR, and 300 million from AAS annually, and these numbers are increasing steadily (Bousquet et al., 2020b). 90 per million males and 170 per million females die from AAS. Low-to-middle income countries report 96% of asthma deaths (Meghji et al., 2021). Current estimates indicate that FA affects 1 to 10 percent of the global population (Kattan and Wang, 2013). The current global prevalence rate of AD is 8% (Tsoi et al., 2020; Lloyd-Lavery et al., 2019), the lifetime prevalence of AD is 20% (Bieber, 2022). In 2019, the number of AD patients worldwide was 171.17 million patients (Dong et al., 2021). Allergic disorders have their origins in a complex interplay between genetic factors and the environment. Although both the innate and adaptive immune systems have important roles in maintaining homeostasis with the environment (Agache and Akdis, 2019), allergies result from a similar interaction of genetic factors and environmental factors, at the phenotypic level, leading to immunological dysregulation at the molecular and cellular levels (Qian et al., 2022; Agache et al., 2019).

Pathogenesis of allergic diseases is considered to involve types I-IV hypersensitivity mechanisms classified by Gell and Coombs, which are often associated with coexisting multiple reaction types. The expression of the disease is determined by the combination of genetic factors, environmental factors, and factors specific to target tissues. The IgE-mediated immune response is critical in the pathogenesis of allergic diseases. As mentioned by Di Lorenzo et al., IgE induces Type I hypersensitivity primarily through its binding to high affinity FcεRI receptors on mast cells and basophils, leading to degranulation and the release of inflammatory mediators (Di Lorenzo et al., 2017). Lambrecht and Hammad wrote: Epithelial damage in the airways results in the release of alarmins that induce the activation of dendritic cells and induce Th2 skewed immunity, which is a prerequisite for asthmatic inflammation (Lambrecht and Hammad, 2015). Akdis suggested: Impaired peripheral immune tolerance may cause chronic allergic inflammation due to the dysfunction of regulatory T cells, which would lead to disease persistence (Akdis, 2012). According to Pawankar, environmental pollutants such as PM2.5 may induce the activation of Toll-like receptor pathways, induce airway hyperresponsiveness, and induce an environment favoring a Th2 shifted shift in immune response (Pawankar, 2014). Warren et al. wrote: Food allergy results from the interaction of genetic susceptibility, intestinal barrier functional defects, and mucosal immune responses (Warren et al., 2021).

Characteristic clinical features: upper respiratory symptoms (sneezing, nasal congestion), lower respiratory symptoms (wheezing, dyspnea), cutaneous manifestations (urticaria, lichenification), and systemic reactions (angioedema, hypotension). Life-threatening conditions are bronchospasm and circulatory collapse. Histamines, proteases, and chemotactic factors released by the mast cells have been identified as the main mediators of increased vascular permeability and smooth muscle contraction in acute anaphylactic shock (Marquardt and Wasserman, 1982). First-generation H1-antihistamines, such as diphenhydramine, have sedative effects due to the central suppression of the nervous system and are commonly associated with anticholinergic side effects like xerostomia, urinary retention (Hossenbaccus et al., 2020). However, according to the pharmacological summary by Ren et al., these drugs can induce severe, rare adverse reactions like hepatotoxicity (Ren et al., 2023). Previous studies have shown that combination antihistamine therapy is an effective method to relieve the acute allergic reaction, and the treatment effect is very remarkable in allergic rhinitis and asthma, etc. However, Atanasio et al. have warned that a subgroup of patients may present with tolerance or decreased therapeutic effect after repeated use for a long time (Atanasio et al., 2022). Therefore, it is very important to find more targeted drugs with individualized therapy. It has been reported that a comprehensive review has indicated that biologic agents have in general a good safety profile, and injection reactions are not frequent, but systemic hypersensitivity events are extremely rare, so close attention should be paid to them (Sitek et al., 2023). At present, the main treatment for allergic diseases is still based on antihistamines, glucocorticoids, leukotriene receptor antagonists, or anti-IgE monoclonal antibodies (Linton et al., 2023; Kumar and Deshmukh, 2024). However, the side effects of these drugs are very common, such as central depression of the nervous system (sedation, attention deficit), anticholinergic effects (xerostomia, dysuria), and hepatorenal toxicity. Therefore, it is very important to find more effective and safer drugs.

Emerging literature has reported that natural products have gained increasing attention in disease intervention owing to their multi-target regulatory characteristics. Natural products induce their pharmacological effects via the regulation of multiple signaling pathways, enzymatic targets, and receptor molecules simultaneously, which endows them with synergistic effects against complex diseases, including cancer, inflammatory, and metabolic diseases (Atanasov et al., 2021; Nasim et al., 2022; Mullowney et al., 2023). In addition to well-studied polyphenols and flavonoids, natural products from other structural classes also exhibit potential bioactivities. For instance, Xiao et al. reported that a polysaccharide-peptide complex exhibits anticancer and immunomodulatory activities mechanistically associated with the modulation of ferroptosis and the TRIM56/STAT3 axis. A large variety of mechanisms of natural products are not exhaustively listed here (Xiao et al., 2025). Quercetin is one of the most studied flavonoid-based natural compounds that exhibits a multitude of pharmacological activities, including anti-inflammatory, antioxidant, and anti-allergic activities, via modulation of signaling pathways, such as NF-κB and MAPK (Li et al., 2016; Chiang et al., 2023; Bernini and Velotti, 2021). In addition, quercetin has been reported to exhibit considerable potential for alleviating metabolic syndrome, diabetes, and tumor growth *in vitro* and *in vivo* studies with considerable efficacy, which deserves further clinical application.

Quercetin is a widely distributed flavonoid compound found in many fruits, vegetables and tea, possesses a variety of pharmacological activities such as immunomodulatory, anti-allergic, antioxidant and anti-inflammatory activities, and shows potential applications in metabolic disorders and cancer suppression. Quercetin is a secondary metabolite of plants and an essential part of human diets (Deepika and Maurya, 2022), Its contents in different plant parts may vary greatly. Therefore, the dietary availability and potential extraction sources of quercetin vary greatly as well. The richest source of quercetin is onion—outer layers and red onions—an edible bulb that can be used as popular vegetables and medicinal agents (Nguyen and Bhattacharya, 2022). In addition to bulbs (Manach et al., 2004), contents of quercetin in leaves and outer layers of many fruits and vegetables are also relatively high. These include skin and outer flesh of many fruits such as apples, dark berries (elderberries, cranberries, lingonberries), cherries, red grapes, citrus fruits (Nemeth and Piskula, 2007), as well as in vegetables including capers, kale, broccoli, and green beans (Deng et al., 2020; Kim et al., 2019). Furthermore, leaves and flowers used for herbal infusions such as tea leaves (Camellia sinensis), Ginkgo biloba leaves, and St. John’s Wort flowers are also rich in quercetin. In addition, it can be found in seeds and grains such as buckwheat, and even in the bark of trees such as the Quercus species (oak).

Bark of some trees such as the Quercus species (oak) has been reported to contain quercetin and its derivatives, however, in lower concentrations when compared with primary edible diet sources such as capers or onions. For example, in the phenolic characterization of Quercus gilvabark, quercetin-3-O-β-D-glucuronide was reported (Docampo-Palacios et al., 2020). Generally, the bark is not the preferred source for commercial or dietary quercetin extraction because of the low yield, environmental impact, and other compounds present such as tannins that interfere with extraction. It should be noted that the concentration of quercetin in any plant part may vary with species, growing conditions, maturity, and post-harvest storage. Nevertheless, current clinical research on quercetin’s anti-allergic effects remains limited by small sample sizes and significant heterogeneity in experimental designs, highlighting the need for systematic evaluation to clarify its translational medical value.

Until now, despite the large number of preclinical studies indicating that quercetin can relieve the symptoms in animal models of allergy, the inconsistent results in different laboratories highlight the importance of comprehensive data mining and analysis. Meta-analysis of preclinical trials is a commonly used method to integrate and analyze animal experimental data, minimize research bias, improve the reliability and translational application of scientific research, and this study will strictly evaluate the possible mechanism of quercetin in animal models of allergy according to Preferred Reporting Items for Systematic Reviews and Meta-Analyses (PRISMA) flow diagram in order to provide scientific basis and theoretical basis for its clinical application.

## Methods

This study adhered to the Preferred Reporting Items for Systematic Review and Meta-Analysis (PRISMA) guidelines and was registered with INPLASY(INPLASY202540003).

### Search strategy

Literature was systematically searched in PubMed, Web of Science, and Embase, from the databases’ establishment to April 2025. Comprehensive inclusion of relevant studies was achieved by supplementing the electronic search with a manual screening of reference lists from eligible articles. The protocol specified the analytical strategy, with serum total IgE, eosinophil count, and IL-4 levels designated as primary outcomes, and other immunological and cytological biomarkers categorized as secondary or exploratory. Supplementary Table S1 offers the detailed search syntax, incorporating keywords and MeSH/Emtree terms.

### Inclusion and exclusion criteria

The study delineated explicit inclusion and exclusion criteria within the PICOTS framework. The study’s inclusion criteria were: participants were limited to animal models affected by allergic diseases; the intervention was the administration of quercetin (aglycone) or its bioactive derivatives. Quercetin glycosides, irrespective of their synthetic or plant-extracted origin, were used in a standardized purity; (3) control groups were assigned to receive either no treatment or an equivalent volume of sterile solution; (4) the primary outcome measures included serum total immunoglobulin E (IgE), ovalbumin-specific IgE (OVA-IgE), cytokine levels (IL-4, IL-5, IL-10, TNF-α, IFN-γ), and immune cell counts (macrophages [Mac], lymphocytes [Lym], eosinophils [Eos], neutrophils [Neu]), as well as histamine (HIS). Supplementary Table S2 provides a detailed account of the PICOTS framework for scholarly reference.

Criteria for exclusion encompassed redundant publications, meta-analyses, review papers, clinical trials, and *in vitro* research. Furthermore, articles without complete text access, research without control groups, and studies without quercetin-based treatment or employing mixed therapeutic approaches with concurrent pharmacological agent use were excluded., such as other flavonoids, antihistamines, or corticosteroids, this factor impedes the identification of quercetin’s unique efficacy; (5) research that lacks focus on allergic disease models; and (6) papers that omitted pre-specified primary results or key data elements., amongst others, flavonoids, antihistamines, or corticosteroids, impeded the identification of quercetin’s unique therapeutic response; (5) research not focused on allergic disease models; and (6) publications that excluded pre-specified primary results or critical data points.

### Data extraction

**FIGURE 1:**
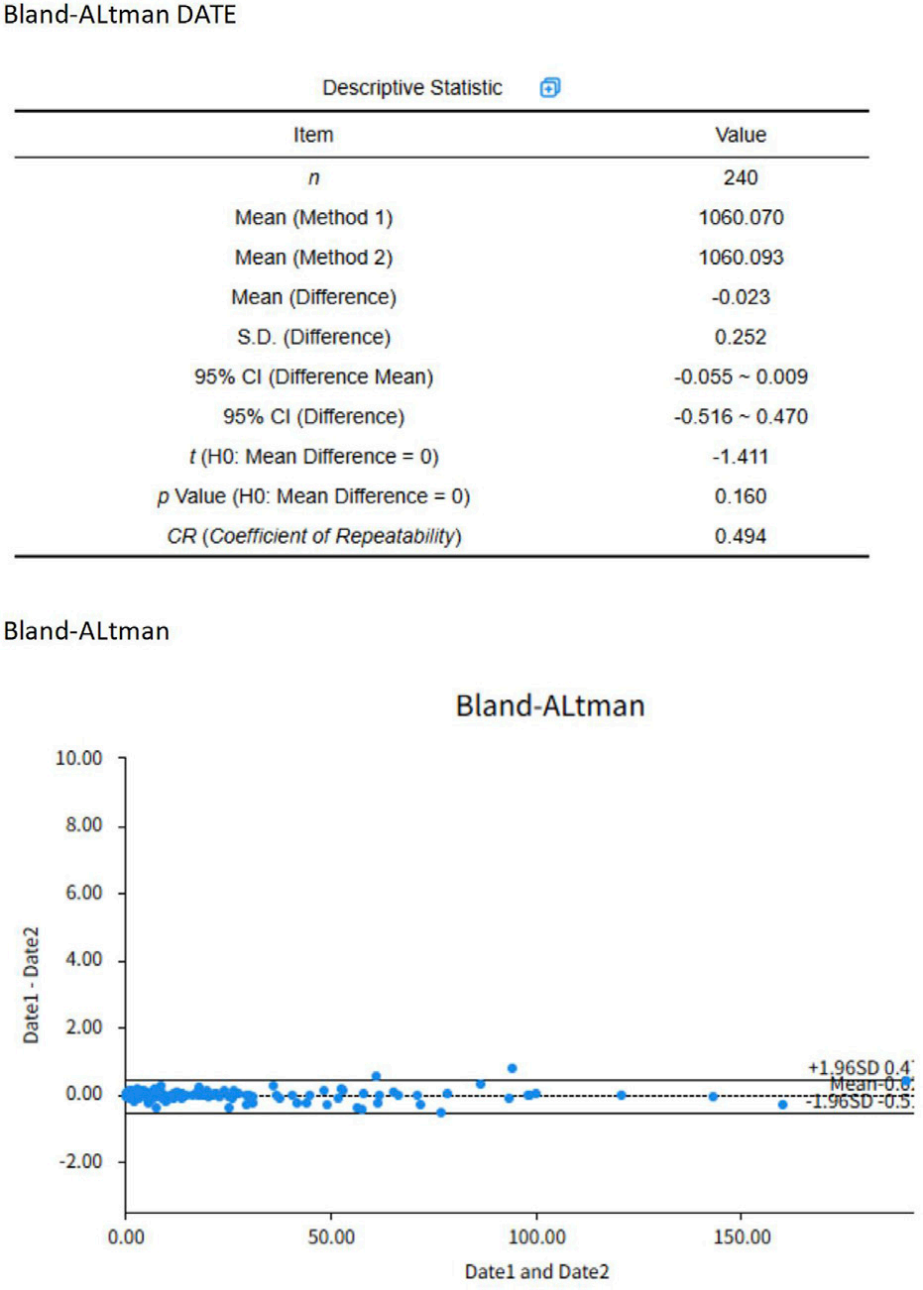
Bland-Altman plot.

The identified studies were systematically managed via NoteExpress software. Following duplicate elimination, Lv and Huang independently conducted a literature search using predefined criteria, with cross-verification performed on all articles meeting the data extraction criteria. The data extraction process entailed the compilation of bibliographic information (title, first author, and publication year), animal characteristics (species, sex, and sample size), modeling methodology (sensitization dose, route of administration, and validation criteria), intervention protocols (administration method, dosage, and duration), and outcome measures. Outcome indicator graphical data were extracted via EngaugeDigitizer graph digitization software, Version 12.1). To ensure the precision of data, a strict error-checking procedure was implemented, involving: (a) the separate digitization of each graph by two researchers; (b) pixel-to-data point calibration based on at least three distinct axis points; (c) cross-validation of extracted values with numerical data from the original articles; and (d) calculation of an intraclass correlation coefficient (ICC) for inter-rater reliability evaluation. Using a two-way mixed-effects model, the ICC was computed from 240 paired comparisons, with absolute agreement as the single measure definition. The ICC estimate, upon computation, was 0. The study yields a value of 998, with a 95% confidence interval (CI) of 0.997 to 0. A rating of 999 signifies impressive dependability Figure 1. In instances of unresolved discrepancies, the corresponding author was engaged in a consultation process for resolution. In instances of unresolved discrepancies, the corresponding author was engaged in a consultation process for resolution.

### Characteristics of included studies

The meta-analysis incorporated 13 preclinical studies, utilizing 183 allergic disease model animals, divided into 89 control subjects and 94 treatment recipients. A multifaceted array of experimental models was utilized in the analysis to thoroughly examine quercetin’s anti-allergic efficacy. A multifaceted array of experimental models was utilized in the analysis to thoroughly examine quercetin’s anti-allergic efficacy.

### Animal models and demographics

The research utilized murine and rat models, with mice forming the majority (128 animals, 67%) as opposed to rats (55 animals, 33%). A spectrum of strains was chosen to replicate allergic conditions, with BALB/c mice used in three studies, C57BL/6 mice in one, SKH-1 hairless mice in one, and NC/Nga mice in one; Wistar and Sprague-Dawley (SD) rat strains were each featured in two studies. Three studies omitted the murine strain detail. The sex distribution data incorporated both male and female animals. Five studies employed female animals exclusively, five utilized male animals exclusively, with the remaining three exhibiting either ambiguous sex reporting or the use of both sexes. The subjects under study were predominantly young adults. Mice exhibited an age range of 6–12 weeks, accompanied by body weights spanning 16–30 g. In experimental studies, rats were generally 8–22 weeks old and weighed between 70 and 250 g, a standard demographic for young adult rodents. In experimental studies, rats were generally 8–22 weeks old and weighed between 70 and 250 g, a standard demographic for young adult rodents.

### Disease models and interventions

The research included a comprehensive selection of seven allergic diseases: asthma (four studies), allergic rhinitis (two studies), food allergy (two studies), atopic dermatitis (two studies), allergic conjunctivitis (one study), allergic airway disease (one study), and anaphylactic shock (one study). The induction of sensitization protocols was mainly achieved through ovalbumin (OVA) solution in ten instances, with other methods including dinitrochlorobenzene (DNCB) with vehicle in one, Dermatophagoides farinae (Df) ointment in one, and chickpea extract (CPE) solution in one. Sensitization protocols predominantly utilized ovalbumin (OVA) solution in ten studies, while alternative methods involved dinitrochlorobenzene (DNCB) with vehicle in one study, Dermatophagoides farinae (Df) ointment in another, and chickpea extract (CPE) solution in a third.

Six studies utilized the aglycone form of quercetin, five used glycoside derivatives (quercitrin and rutin), and one featured a novel covalent conjugate. Ten studies favored intraperitoneal injection as the primary route of administration, with two studies employing topical application on the dorsal skin. Dosing regimens exhibited substantial variability, with quercetin aglycone equivalent doses initiating at 1.11–100 mg/kg. Treatment durations exhibited substantial diversity, with durations ranging from 14 days for short-term to 60 days for long-term interventions. Treatment durations exhibited substantial diversity, with durations ranging from 14 days for short-term interventions to 60 days for long-term regimens.

Outcome Measures and Methodological Quality Outcome measures were assessed by analysing the following immunological biomiomarkers: immunoglobulin profiles [total IgE in n = 8; OVA-specific IgE in n = 5], cytokine levels [IL-4 in n = 7; IL-5 in n = 5; IL-10 in n = 3; TNF-α in n = 6; IFN-γ in n = 4], number of inflammatory cells in bronchoalveolar lavage fluid or tissue [eosinophils [Eos] in n = 7; macrophages [Mac]; lymphocytes [Lym]; neutrophils [Neu] collectively in n = 4] and histamine (HIS) quantification in n = 3 studies. The outcome of interest was assayed from different sample matrices [serum; BALF; tissue homogenates]. All n = 13 studies reported the use of randomisation when allocating study participants into groups. Implementation of blinding of outcome assessment was less consistently reported [n = 5 studies confirmed implementation of blinding of outcome assessment]; the majority of studies were rated as “Unclear” in this domain [n = 7 studies].

### Risk bias evaluation

Two independent reviewers evaluated the studies founded on the inclusion and exclusion criteria and there were no disagreements between the two reviewers on the selection of articles for inclusion.

The quality of the methodological aspects of the included studies were evaluated by the SYRCLE’s Risk of Bias Tool (Systematic Review Center for Laboratory Animal Experimentation’s RoB Tool) (Hooijmans et al., 2014) This tool assesses 10 aspects that are important for bias detection in animal experiments and rates them as follows: random sequence generation; baseline characteristics comparability; allocation concealment; random housing; blinding of caregivers/investigators; random outcome assessment; blinding of outcome assessors; completeness of outcome data; selective outcome reporting; other confounding factors. These 10 aspects were graded as “yes” (low risk of bias), “no” (high risk of bias), or “uncertain” (unclear risk of bias). Discrepancies were resolved through group discussions or by the corresponding authors.

### Statistical analysis

Statistical analysis was performed using Review Manager (RevMan, Version 5.4). Continuous variables were analyzed as primary outcome measures. We computed summary statistics as standardized mean differences (SMD) with corresponding 95% confidence intervals (95% CI) to standardize effect estimates across studies with different measurement scales. A pre-specified strategy was used to include each study only once in the primary meta-analysis, to ensure statistical independence across the included studies. When studies reported multiple quercetin intervention arms (e.g., different quercetin doses or derivatives), we selected the arm with highest dose of quercetin aglycone equivalent for synthesis. Among the 13 included studies, 3 studies (Park et al., 2009; Ding et al., 2020; Chen and Ko, 2021) satisfied the rule of selecting a single high-dose arm. Arm-combining was not performed in the primary analysis, to avoid introducing heterogeneity from different dose-response relationships. The final list of detailed handling for each multi-arm study is presented in Supplementary Table S3.

When the standard deviations (SDs) were not reported, we followed the following hierarchy of imputation: (1) if standard errors of the mean (SEM) were reported, SD was calculated as SD = SEM × √(n); (2) if 95% CIs were reported, SD was calculated as SD = √(n) × (upper limit–lower limit)/3.92; (3) if only interquartile ranges (IQR) or ranges were reported, SD and mean were calculated using validated methods (Hozo et al., 2005). All conversions are explicitly documented in the Supplementary Materials. If means were extracted from logarithmic axes, or if unit conversion was necessary, appropriate mathematical transformations were applied and documented. Between-study heterogeneity was evaluated using the I-squared (I^2^) statistic, which represents the percentage of total variability in effect sizes due to true inter-study heterogeneity (rather than random chance). Given the considerable degree of heterogeneity (I^2^ > 50%) caused by variations in experimental parameters, including murine strains (e.g., BALB/c vs. C57BL/6), allergen types (e.g., ovalbumin vs. DNCB), and administration routes (oral gavage vs. intraperitoneal injection), we chose a random-effects model to account for this heterogeneity. Subgroup analyses and *post hoc* sensitivity analyses were applied to explore potential sources of heterogeneity.

## Results

### Study selection

**FIGURE 2:**
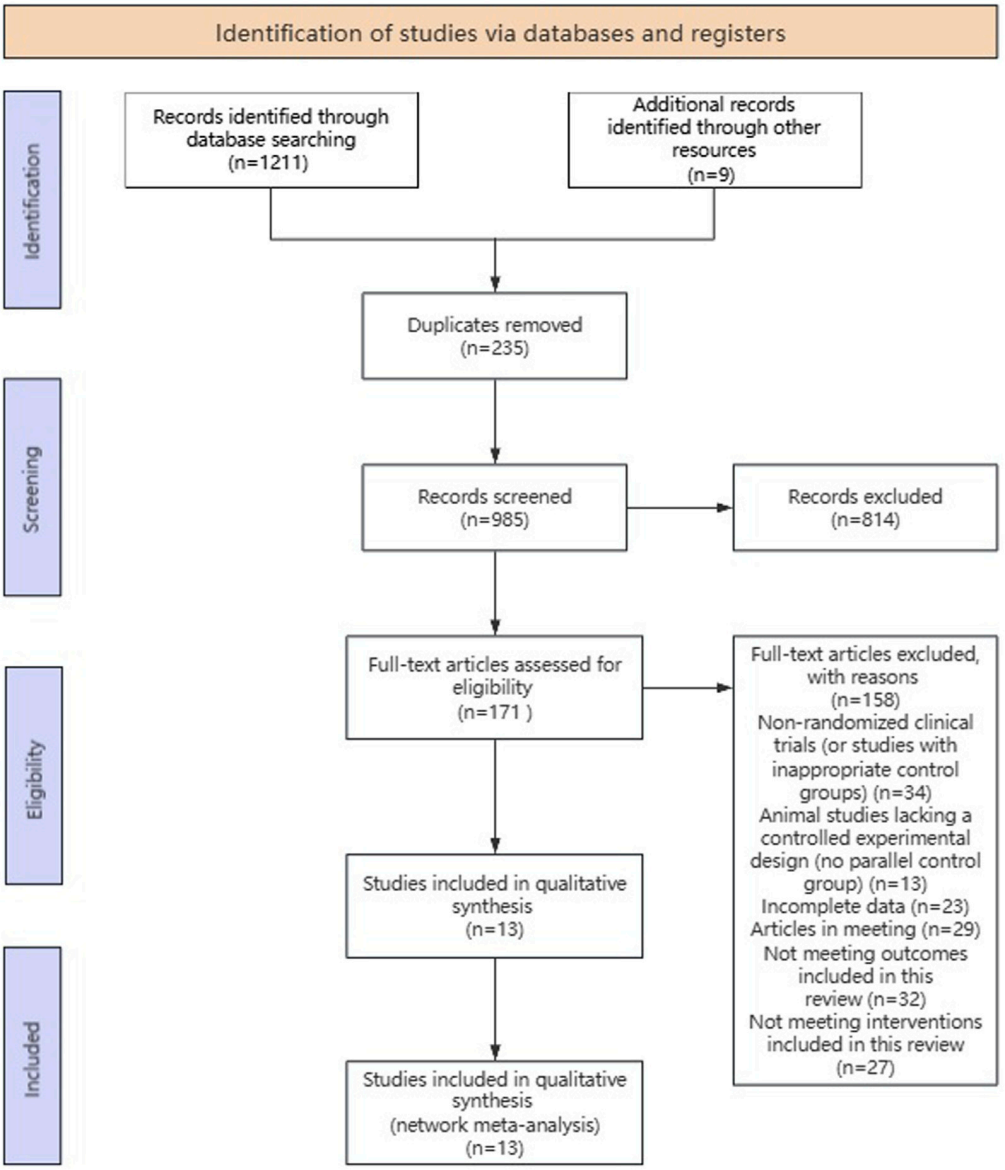
Flow chart of study selection.

Study selection summary flow diagram depicted in Figure 2. A total of 1,211 records were identified from searching the three databases and 9 studies were obtained from other sources, yielding a combined total of 1,211 records. These records were deduped to 985 unique records. These 985 records were title/abstract screened to identify potentially eligible studies, of which 171 were potentially eligible. These potentially eligible studies were further evaluated by checking the full text, and 158 studies were excluded for the following reasons: Not Randomized Clinical Trials (or inappropriate control group); Animal Studies (lack controlled experimental design, not a parallel control group); incomplete/inaccessible data; conference abstracts or non-peer reviewed publications; outcome not relevant to scope of review; intervention not within inclusion criteria; duplicate dataset. Thirteen studies (Cruz et al., 2008; Cruz et al., 2012; Park et al., 2009; Park et al., 2015; Shishehbor et al., 2010; Sagit et al., 2017; Cai et al., 2017; Ding et al., 2020; Jegal et al., 2020; Chen and Ko, 2021; Rajizadeh et al., 2023; Zhou et al., 2024; Mu et al., 2024) met all eligibility criteria upon full-text assessment and were included in the meta-analysis.

### Characteristics of included studies

The meta-analysis included 13 studies with a total of 183 allergic disease model animals, including 89 model control groups and 94 treatment groups. A total of 128 mice (67%) and 55 rats (33%) were used. The specific strains of mice are as follows: 3 studies used BALB/c mice, 1 study used SKH-1 hairless mice, 1 study used C57BL/6 mice, 1 study used NC/Nga mice, and 3 studies did not specify the murine strain; 2 studies each used Wistar rats and Sprague-Dawley (SD) rats. The stratification of disease models is shown in Supplementary Table S3: asthma, 4 studies; allergic rhinitis, 2; food allergy, 2; atopic dermatitis, 2; allergic conjunctivitis, 1; allergic airway disease, 1; and anaphylactic shock, 1. The most commonly used sensitization protocol was ovalbumin (OVA) solution (10 studies); other approaches included DNCB with vehicle (1 study), Dermatophagoides farinae (Df) ointment (1 study), and chickpea extract (CPE) solution (1 study). The main immunological biomarkers examined were immunological indicators related to immunological mechanisms: immunoglobulin profiles (IgE in 8 studies, OVA- IgE in 5 studies), cytokine levels (IL-4 in 7 studies, IL-5 in 5 studies, IL-10 in 3 studies, TNF-α in 6 studies, IFN-γ in 4 studies), inflammatory cell counts (Eos in 7 studies; Mac, Lym, and Neu in 4 studies collectively), and HIS in 3 studies. Detailed descriptions of the overall characteristics of the studies are shown in Supplementary Table S3.

### Quality assessment

**FIGURE 3:**
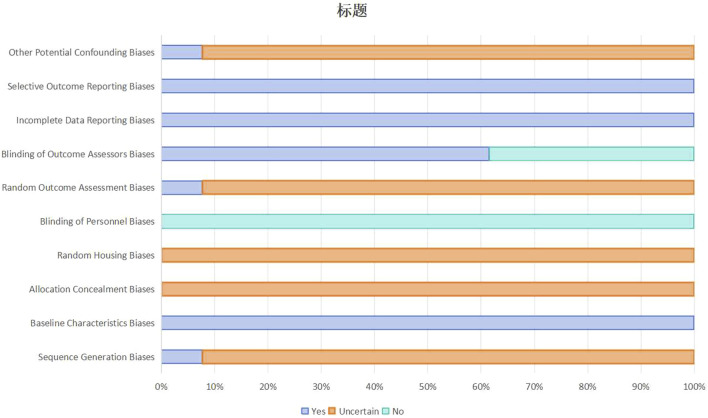
Summary of risk of bias assessment for included studies.

Methodological quality of the included studies using the methodological quality assessment tool developed by SYRCLE (Hooijmans et al., 2014), are summarised in Figure 3 and Supplementary Table S4. All 13 studies were at low risk of bias for selective outcome reporting, completeness of outcome data and comparability of baseline characteristics. Blinding of outcome assessors to allocate allocation was performed in 8 studies (61.5%). However, there were methodological gaps with respect to randomization: documentation of random housing protocols to reduce the risk of environmental confounding was not reported in any of the studies, allocation concealment during group allocation was not explicitly described in any of the studies, and only one study (7.7%) was rated as “Low Risk” for random sequence generation. This study reported using a computer-generated random number table (Chen et al., 2021). The remaining 12 studies (92.3%) were rated “Unclear” with respect to random sequence generation due to inadequate description of the sequence generation process.

## Conclusions of the meta-analysis

### Primary outcomes

**FIGURE 4:**
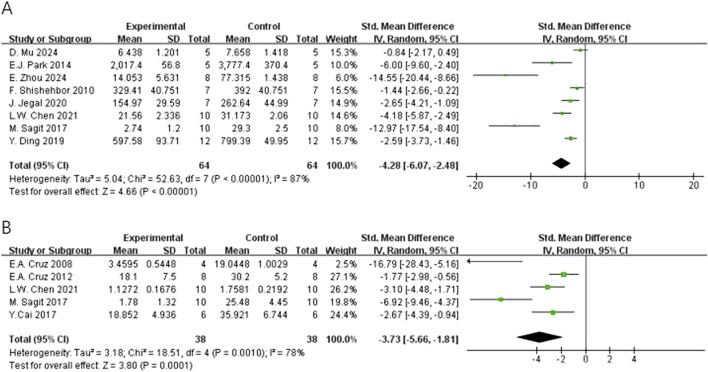
Forest plot of the effect of quercetin on **(A)** total IgE, **(B)** OVA-IgE.

**FIGURE 5:**
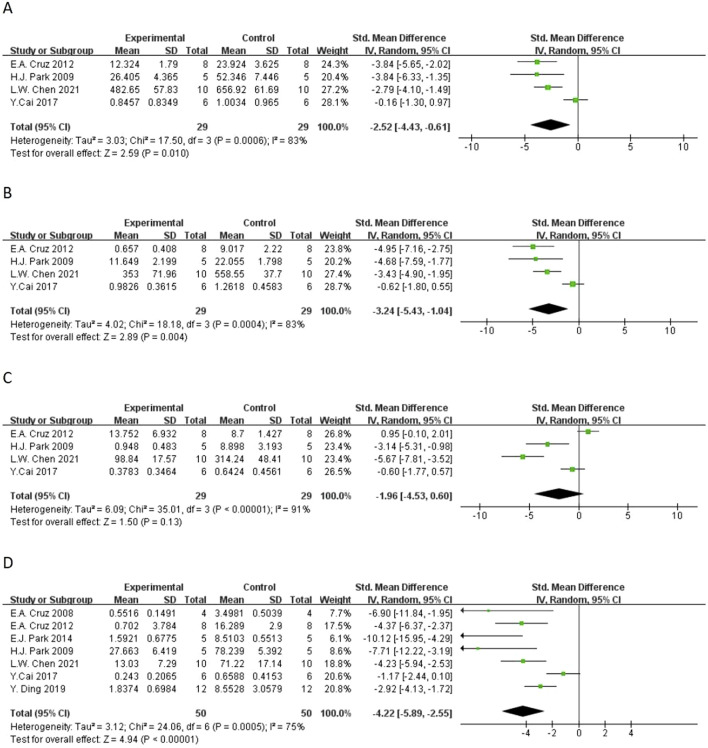
Forest plot of the effect of quercetin on **(A)** macrophage counts, **(B)** lymphocyte counts, **(C)** neutrophil counts, **(D)** eosinophil counts.

**FIGURE 6:**
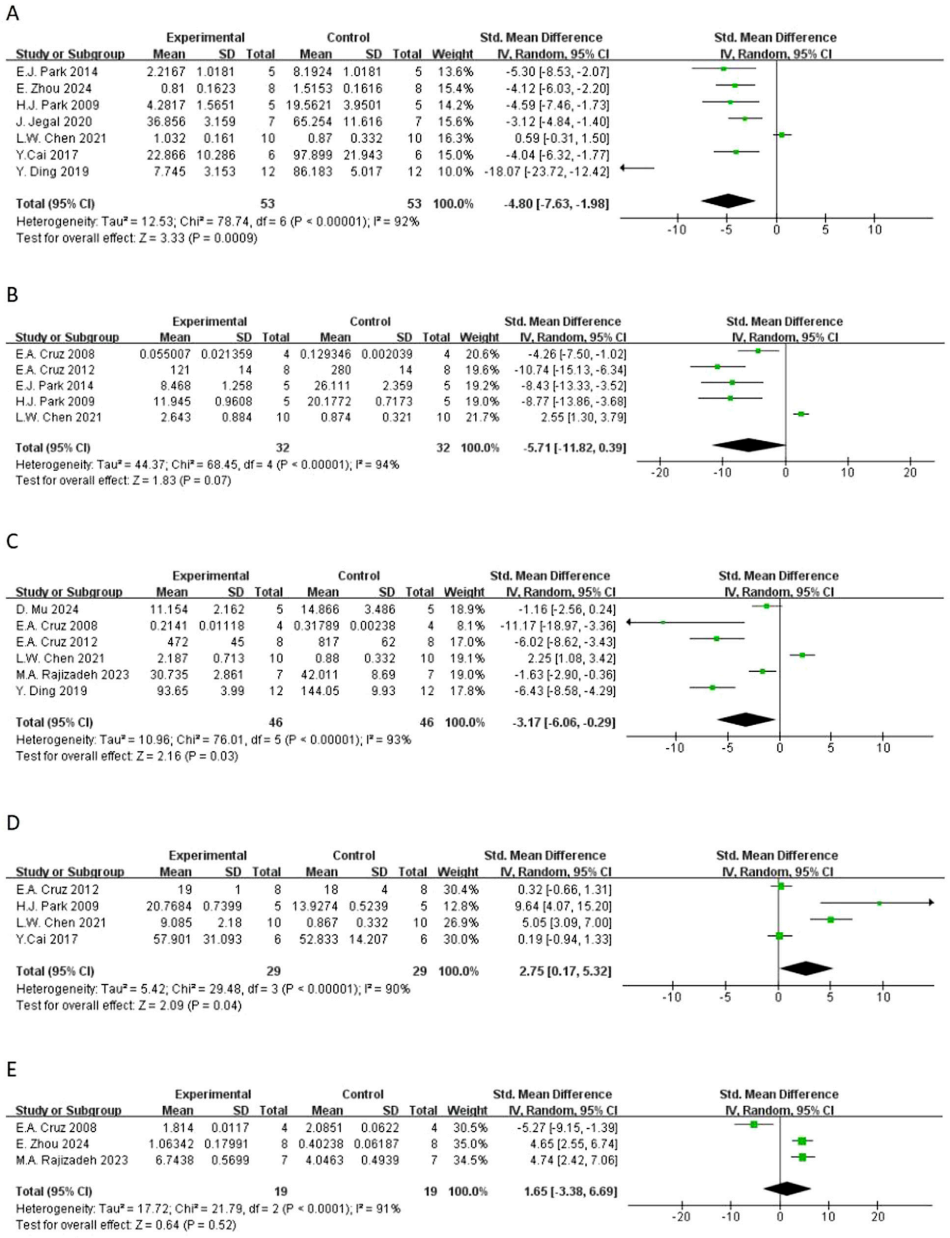
Forest plot of the effect of quercetin on **(A)** IL-4, **(B)** IL-5, **(C)** TNF-α, **(D)** IFN-γ, **(E)** IL-10.

In regard to primary outcomes, the result showed that quercetin had regulatory effects. In terms of the modulation of immunoglobulin E (IgE), the result of the meta-analysis based on eight studies demonstrated that quercetin significantly decreased the level of total IgE [n = 8, SMD = −4.28 (95% CI: –6.07 to −2.48), p < 0.00001, I^2^ = 87%] (Figure 4A). In regard to inflammatory cells, the result of the meta-analysis based on seven studies demonstrated that quercetin significantly decreased the level of eosinophils [n = 7, SMD = −4.22 (95% CI: –5.89 to −2.55), p < 0.00001, I^2^ = 75%] (Figure 5D). At the cytokine level, the result of the meta-analysis based on seven studies demonstrated that quercetin significantly decreased the expression of interleukin-4 (IL-4), an important cytokine secreted by Th2 cells [n = 7, SMD = −4.80 (95% CI: –7.64 to −1.98), p = 0.0009, I^2^ = 92%] (Figure 6A). These three key indicators showed that quercetin significantly inhibited the core allergic process.

### Secondary outcomes

In terms of secondary outcomes, quercetin also displayed multi-target regulatory potential. With regard to particular immune response, five studies reported that quercetin could significantly decrease the content of ovalbumin-specific IgE (OVA-IgE) [n = 5, SMD = −3.73 (95% CI: –5.66 to −1.81), p = 0.0001, I^2^ = 78%] (Figure 4B). As for rebalancing of cytokines, six studies reported that quercetin could significantly decrease the content of TNF-α [n = 6, SMD = −3.17 (95% CI: –6.06 to −0.29), p = 0.03, I^2^ = 93%] (Figure 6C), while four studies reported that quercetin could significantly increase the content of IFN-γ [n = 4, SMD = 2.75 (95% CI: 0.17–5.32), p = 0.04, I^2^ = 90%] (Figure 6D), which indicated that quercetin could induce the rebalancing of Th1/Th2 immune balance toward Th1 phenotype. In addition, as for immune cell subsets, the content of macrophage was significantly decreased by quercetin [n = 4, SMD = −2.52 (95% CI: –4.43 to −0.61), p = 0.010, I^2^ = 83%] (Figure 5A) and the content of lymphocyte was significantly increased [n = 4, SMD = −3.24 (95% CI: –5.43 to −1.04), p = 0.004, I^2^ = 83%] (Figure 5B), which further demonstrated the wide immunomodulatory effect of quercetin.

### Exploratory outcomes

In the exploratory analysis, some outcomes showed significant trends, but were not statistically significant.

**FIGURE 7:**
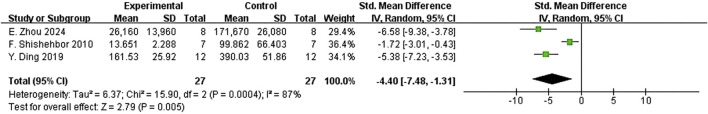
Forest plot of the effect of quercetin on HIS.

Interleukin-5 (IL-5) had a downward trend that was not statistically significant [n = 5, SMD = −5.71 (95% CI: –11.82 to 0.39), p = 0.07, I^2^ = 94%] (Figure 65B). The variations in the anti-inflammatory cytokine IL-10 were not significant [n = 3, SMD = 1.65 (95% CI: –3.38–6.69), p = 0.52, I^2^ = 91%] (Figure. Neutrophil count also had a reduction trend that was not statistically significant [n = 4, SMD = −1.96 (95% CI: –4.53 to 0.60), p = 0.13, I^2^ = 91%] (Figure 5C). Interestingly, three studies demonstrated that quercetin could significantly decrease histamine (HIS) level [n = 3, SMD = −4.40 (95% CI: –7.48 to −1.31), p = 0.005, I^2^ = 87%] (Figure 7).

### Subgroup analysis

Because of the large heterogeneity we detected among studies, we further conducted the subgroup analyses according to the following methodological and experimental factors: administration route, treatment duration, induction model, sample matrix, animal species, and disease type to explore the possible sources of the variation for multiple immunological outcomes.

Subgroup analysis according to administration route, results showed that ip quercetin significantly decreased the total IgE [n = 6, SMD = −4.56 (95% CI: –6.83 to −2.28), p < 0.0001, I^2^ = 90%], OVA-IgE [n = 4, SMD = −3.34 (95% CI: –5.03 to −1.65), p = 0.0001, I^2^ = 77%], macrophage count [n = 4, SMD = −2.52 (95% CI: –4.43 to −0.61), p = 0.01, I^2^ = 83%], lymphocyte count [n = 4, SMD = −3.24 (95% CI: –5.43 to −1.04), p = 0.004, I^2^ = 83%], eosinophil count [n = 5, SMD = −3.48 (95% CI: –5.05 to −1.90), p < 0.0001, I^2^ = 75%], and IL-4 level [n = 5, SMD = −5.29 (95% CI: –9.21 to −1.37), p = 0.008, I^2^ = 94%]. Other administration routes also exhibited the significant decreases in the total IgE, OVA-IgE, eosinophils, IL-4, IL-5, IL-10, and TNF-α, however, with the smaller sample size and sometimes non-significant heterogeneous outcomes (Supplementary Table S5).

When stratified by treatment duration, short-term treatment (<30 days) markedly decreased total IgE [n = 3, SMD = −9.76 (95% CI: –18.41 to −1.11), p = 0.03, I^2^ = 93%], OVA-IgE [n = 3, SMD = −6.17 (95% CI: –10.82 to −1.52), p = 0.009, I^2^ = 83%], IL-5 [n = 2, SMD = −6.07 (95% CI: –10.40 to −1.74), p = 0.006, I^2^ = 53%] and histopathology scores [n = 1, SMD = −6.58 (95% CI: –9.38 to −3.78), p < 0.00001]. Long-term treatment (≥30 days) significantly decreased total IgE [n = 5, SMD = −2.57 (95% CI: –3.92 to −1.21), p = 0.0002, I^2^ = 74%], OVA-IgE [n = 2, SMD = −2.39 (95% CI: –3.68 to −1.10), p = 0.0003, I^2^ = 50%], macrophages [n = 2, SMD = −3.15 (95% CI: –4.21 to −2.09), p < 0.00001, I^2^ = 0%], lymphocytes [n = 2, SMD = −3.96 (95% CI: –5.38 to −2.53), p < 0.00001, I^2^ = 21%], eosinophils [n = 4, SMD = −4.15 (95% CI: –5.69 to −2.60), p < 0.00001, I^2^ = 57%] and IL-10 [n = 1, SMD = 4.74 (95% CI: 2.42–7.06), p < 0.0001]. Of note, the effect sizes induced by short-term interventions in general were larger than those induced by long-term treatments (Supplementary Table S6).

Subgroup analysis based on induction model showed that OVA-induced models had significant decreases in total IgE [n = 5, SMD = −5.70 (95% CI: –8.67 to −2.72), p = 0.0002, I^2^ = 91%], OVA-IgE [n = 5, SMD = −3.73 (95% CI: –5.66 to −1.81), p = 0.0001, I^2^ = 78%], eosinophils [n = 6, SMD = −3.74 (95% CI: –5.30 to −2.18), p < 0.00001, I^2^ = 73%] and TNF-α [n = 6, SMD = −3.17 (95% CI: –6.06 to −0.29), p = 0.03, I^2^ = 93%]. DNCB-induced and Df-induced models also exhibited significant decreases in IgE, IL-4 and IL-5, based on few studies (Supplementary Table S7).

Analysis according to sample matrices revealed that quercetin could significantly decrease IgE content in serum [n = 8, SMD = −4.28 (95% CI: –6.07 to −2.48), p < 0.00001, I^2^ = 87%]. Eosinophils number was decreased in serum [n = 2, SMD = −8.24 (95% CI: –12.01 to −4.47), p < 0.0001, I^2^ = 0%], tissue [n = 2, SMD = −4.80 (95% CI: –9.38 to −0.22), p = 0.04, I^2^ = 75%], and BALF [n = 3, SMD = −3.17 (95% CI: –5.43 to −0.91), p = 0.006, I^2^ = 82%]. The expression of cytokines including IL-4, IL-5 and TNF-α were also significantly suppressed in serum and tissue samples (Supplementary Table S8).

When aggregated by animal type, mice exhibited significant decreases in total IgE [n = 7, SMD = −4.67 (95% CI: –8.81 to −0.53), p = 0.03, I^2^ = 94%], OVA-IgE [n = 3, SMD = −2.97 (95% CI: –5.31 to −0.62), p = 0.01, I^2^ = 75%], macrophages [n = 3, SMD = −3.25 (95% CI: –4.23 to −2.28), p < 0.00001, I^2^ = 0%], lymphocytes [n = 3, SMD = −4.02 (95% CI: –5.15 to −2.89), p < 0.00001, I^2^ = 0%], and IL-4 [n = 6, SMD = −5.01 (95% CI: –8.25 to −1.77), p = 0.002, I^2^ = 93%]. Rats exhibited effects significant only for OVA-IgE [n = 2, SMD = −4.68 (95% CI: –8.84 to −0.52), p = 0.03], TNF-α [n = 1, SMD = −1.63 (95% CI: –2.90 to −0.36), p = 0.01], and HIS scores [n = 1, SMD = −1.72 (95% CI: –3.01 to −0.43), p = 0.009] (Supplementary Table S9).

Subgroup analysis according to disease models showed that quercetin markedly decreased total IgE in atopic dermatitis [n = 2, SMD = −3.92 (95% CI: –7.10 to −0.73), p = 0.02, I^2^ = 64%] and other allergic models [n = 2, SMD = −3.26 (95% CI: –4.79 to −1.73), p < 0.0001, I^2^ = 57%]. OVA-IgE was markedly decreased in asthma [n = 2, SMD = −2.93 (95% CI: –4.01 to −1.85), p < 0.00001, I^2^ = 0%] and allergic rhinitis models [n = 1, SMD = −6.92 (95% CI: –9.46 to −4.37), p < 0.00001]. Eosinophil counts were significantly decreased in asthma [n = 3, SMD = −3.78 (95% CI: –6.85 to −0.72), p = 0.02, I^2^ = 85%] and other models [n = 4, SMD = −4.80 (95% CI: –7.06 to −2.54), p < 0.0001, I^2^ = 64%]. IL-4 levels were decreased in asthma [n = 2, SMD = −3.77 (95% CI: –5.71 to −1.82), p = 0.0001, I^2^ = 26%], and IL-5 in non-asthma models [n = 3, SMD = −7.56 (95% CI: –11.65 to −3.47), p = 0.0003, I^2^ = 66%]. IL-10 was increased in asthma and food allergy models but reduced in others (Supplementary Table S10).

### Visual summary of subgroup analyses via forest plots

To offer an intuitive synthesis of our subgroup findings, we have added six summary forest plots in Supplementary Table S11, representing six stratification variables: animal species, disease model, induction method, sample matrix, administration route, and treatment duration respectively. These forest plots visually summarize the pooled SMDs, 95% CIs, I^2^ estimates, and statistical significances of each subgroup, which allow direct comparison of quercetin’s effectiveness across experimental conditions.

The forest plots demonstrate the similar effectiveness of quercetin for different allergic phenotypes despite experimental variations. For example, the summary plot of animal species shows that quercetin exhibited a larger reduction in IgE and cellular infiltration in mice than in rats, which is in line with quantitative subgroup results. Likewise, the disease model plot shows consistent quercetin suppression of IgE in atopic dermatitis and asthma models, supporting the translational relevance of quercetin across allergic phenotypes.

For full methodological transparency and granularity, the detailed forest plots of 72 subgroups are available in Supplementary Materials.

This visual integration of results complement the tabular results and facilitate the interpretation of subgroup findings, bridging quantitative synthesis with clinical and experimental results.

### Publication bias and sensitivity analysis

This study adhered to the recommendations of systematic reviews and meta-analyses and employed Begg’s rank correlation test and Egger’s linear regression test to evaluate publication bias of 13 included preclinical trials (Supplementary Table S12). The aim was to detect potential small-study effects and selective publication bias. It is noteworthy that Begg’s and Egger’s tests exhibit low sensitivity and obtain unreliable conclusions when the number of studies is small (k < 10) (Sterne et al., 2011). Therefore, the results of these tests for several outcomes in this study (IL-5, k = 5; IL-10, k = 3) should be interpreted with extreme caution and only used as auxiliary references rather than definitive conclusions. The test results showed heterogeneous publication patterns of different immune indicators during evidence synthesis.

Statistically significant publication bias (p < 0.05) was found for four indicators: total IgE (Begg’s Test p = 0.026; Egger’s Test p = 0.003), eosinophils (EOS) (Begg’s Test p = 0.024; Egger’s Test p = 0.018), IL-4 (Egger’s Test p = 0.001), and IL-5 (Egger’s Test p = 0.002). These results indicated that the effect sizes of these indicators in the meta-analysis might be overestimated. However, no publication bias was found for OVA-IgE, macrophages, lymphocytes, neutrophils, IL-10, TNF-α, or histamine (HIS) (the p-value of both Begg’s and Egger’s tests were >0.05), which enhanced the reliability of our results.

Additionally, according to the suggestion of the reviewer, we carried out trim-and-fill analyses to further explore the potential impact of publication bias. It is important to note that because of the limited number of included studies and high heterogeneousness, these trim-and-fill analyses are only exploratory. The results of trim-and-fill analyses should be treated as sensitivity analysis of the tool to explore the potential impact of publication bias, and the results of trim-and-fill analyses should be interpreted cautiously.

This systematic review carried out a comprehensive meta-analysis of primary outcome measures (IgE and OVA-IgE), and assessed the robustness of the results and quality of evidence before and after sensitivity analysis.

The initial analysis (Figure 4) showed that 8 studies were included for total IgE, and the pooled effect size was Standardized Mean Difference (SMD) = −6.36 (95% CI: -9.62 to −3.11, P < 0.00001), with significant heterogeneousness (I^2^ = 87%, Tau^2^ = 5.04). For OVA-IgE, 5 studies were included, and the pooled SMD = −4.01 (95% CI: -6.40 to −1.63, P = 0.001), with significant heterogeneousness (I^2^ = 78%, Tau^2^ = 3.18).

**FIGURE 8:**
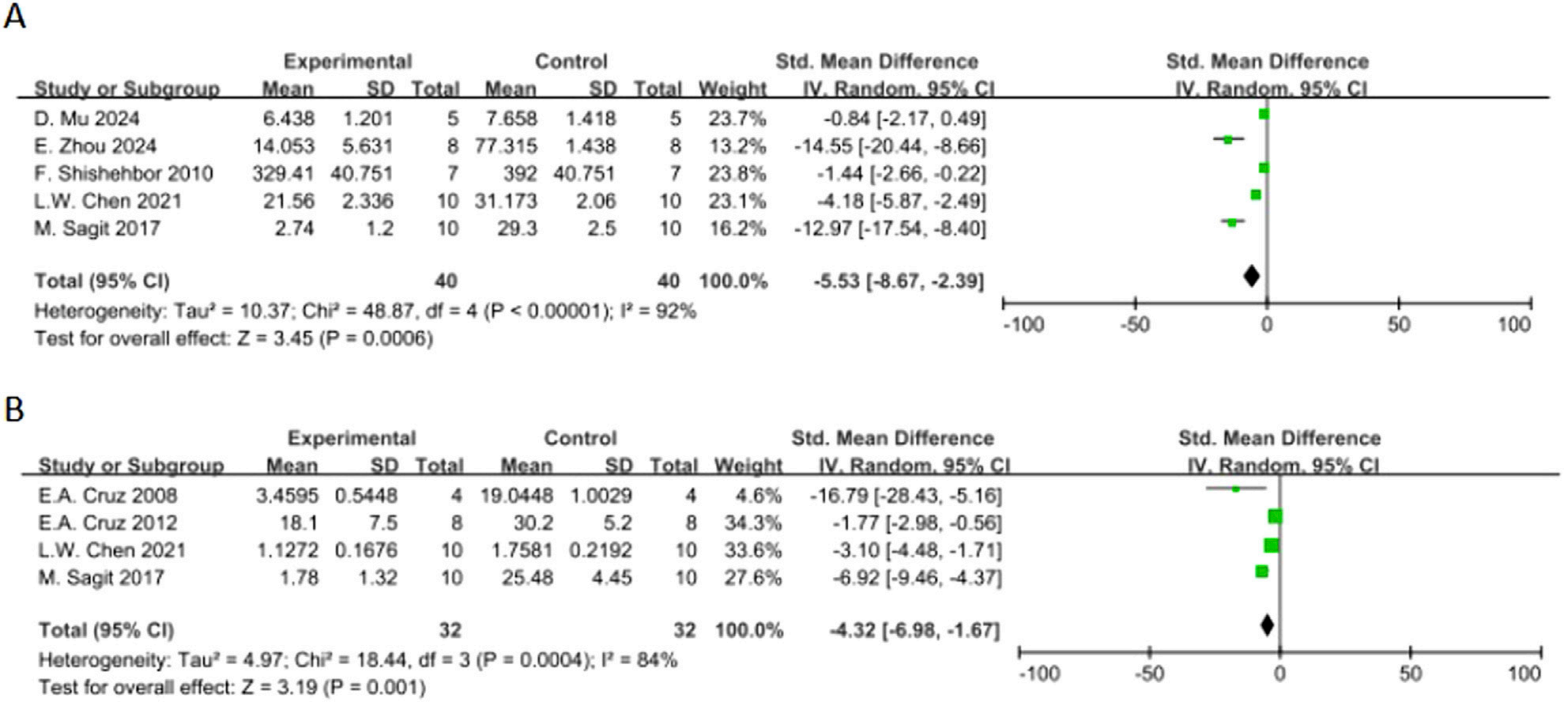
Forest plot of the effect of quercetin on **(A)** total IgE, **(B)** OVA-IgE in the sensitivity analysis.

Sensitivity analysis was pre-specified and aimed to investigate the effect of potential bias on results of these studies [14] by excluding 4 studies with low RoB Score (RoB Score = 10) (Park et al., 2015; Jegal et al., 2020; Ding et al., 2020; Cai et al., 2017). Post-hoc analysis (Figure 8) revealed that, for total IgE (5 studies), SMD changed to SMD = −5.53 (95% CI: -8.67 to −2.39, P = 0.0006) and for OVA-IgE (4 studies), SMD changed to SMD = −4.32 (95% CI: -6.98 to −1.67, P = 0.001). After sensitivity analysis, the direction of effects and statistical significance of both outcome measures were not changed.

## Discussion

Our systematic review and meta-analysis of 13 preclinical studies suggests that quercetin has a beneficial impact on the control of allergic disease in animal models. The quantitative synthesis showed that quercetin treatment improved immunological parameters significantly, with quercetin treatment significantly decreasing the levels of total IgE, OVA-specific IgE, and histamine compared with the disease control. The compound exhibited strong immunomodulatory effects, significantly reducing inflammatory cell infiltration, as shown by the significant decreases in the numbers of macrophages, lymphocytes, eosinophils, and neutrophils. At the molecular level, quercetin significantly decreased the concentration of pro-inflammatory cytokines (IL-4; IL-5; TNF-α) and increased the level of anti-inflammatory IFN-γ, indicating that quercetin exerts dual effects on the regulation of Th2-mediated inflammatory and oxidative stress pathways Notably, we found significant heterogeneity among the primary outcomes between studies. The sources of heterogeneity may be related to the designs of the experiments, the methods of dosing, and animal models used in the studies.

**FIGURE 9:**
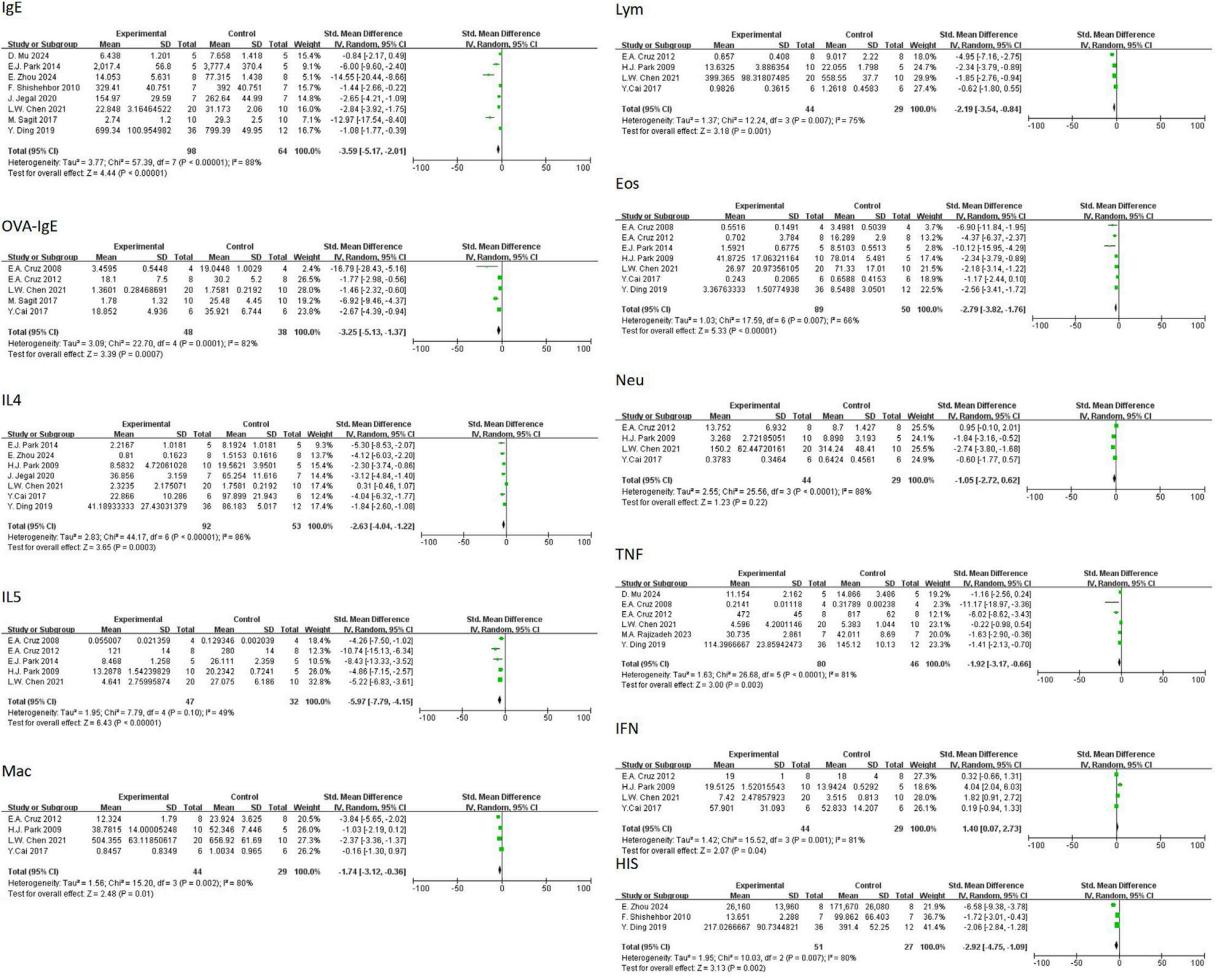
Post-hoc sensitivity analysis.

In addition, we would like to make a note about how we handled multi-arm studies. Our decision to select only the highest dose for the primary analysis, instead of combining arms, was conservative because it served to maintain independence among data points and offered a clear, interpretable estimate of the maximal potential effect. However, our analysis of the primary outcome using all arms (*post hoc* sensitivity analysis, see Figure 9) where we deliberately forced the analysis to include all available dose arms, did show changes in effect sizes and heterogeneity. Since dose is clearly a major source of variation, we justified our initial strategy of not combining pharmacologically different interventions and only selecting the highest dose available.

### Primary outcomes

This is the first comprehensive analysis of quercetin’s suppressive effects on therapeutic mechanisms of allergic responses. In this study, we further investigated quercetin’s regulation on immunoglobulins and inflammatory mediators.

Meta-analysis of eight studies on the total IgE were included. Quercetin significantly suppressed the total IgE levels. IgE is a central mediator in the pathogenesis of allergic diseases. It plays a critical role in type I hypersensitivity reactions, which are involved in approximately one third of the world population (Mu et al., 2024). The suppression of quercetin on the IgE production demonstrated its potential as immunomodulatory agent for the allergic diseases.

The recent work by Park et al. has demonstrated marked downregulation of serum IgE in an atopic dermatitis model (Park et al., 2015) New pharmacological entities have been developed based on quercetin by Zhou et al. (2024), The quercetin–conjugated formulation exhibits more effective IgE suppression by covalent modification. The results of Mu et al. that indicated quercetin possesses multi-targets anti-inflammatory activity also support this finding (Mu et al., 2024).

Quercetin exerted significant effects on eosinophil-induced inflammation. Pooled analysis of seven studies revealed a considerable decrease in the eosinophil levels. Eosinophils play a critical role in allergic asthma models. OVA-induced allergic asthma models have been widely used. It is well established that eosinophil infiltration was a typical characteristic of asthma pathology. Quercetin significantly suppressed eosinophil peroxidase activity, tissue migration and bronchial remodeling induced by asthma. The results of Cruz et al. (2012), Cai et al. (2017), and Ding et al. (2020) were consistent with ours. Moreover, derivatives of quercetin exhibited cell-selective suppression on eosinophils, neutrophils and lymphocytes without compromising macrophage function.

Quercetin significantly suppressed the expression of Th2 cytokine IL-4. It is well known that IL-4 induced IgE class switching and mast cell proliferation. Jegal et al. (2020), Ding et al. (2020), and Park et al. (2009), Park et al. (2015) in our previous work has demonstrated the inhibition of IL-4 secretion by quercetin.

### Secondary outcomes

In addition, quercetin repeatedly suppressed allergen-specific immune pathways in secondary outcomes. OVA-IgE five studies reported that quercetin markedly suppressed allergen-specific IgE, a mechanism initially discovered in the study by Cruz et al. (2012) and further verified in subsequent studies by Cai et al. (2017) and Sagit et al. (2017). Chen and Ko (2021) also found that quercetin inhibited total IgE and OVA-IgE in systemic and local compartments, indicating quercetin’s potential application in all allergic phenotypes.

Quercetin significantly modulated cellular immunity, reducing macrophage and lymphocyte counts. The multi-cell modulation of quercetin—targeting macrophages, lymphocytes and eosinophils—demonstrates that quercetin can attenuate the activation of innate and adaptive immunity in allergic conditions.

Rebalancing of cytokines was another important secondary outcome. Quercetin significantly suppressed TNF-α, a pro-inflammatory cytokine that induces endothelial activation and airway hyperreactivity (Mu et al., 2024),while increasing IFN-γ, a Th1 cytokine. The increase in TNF-α and IFN-γ levels in studies such as Ding et al. (2020) and Cruz et al. (2012), reflect a Th1-biased response, indicating that quercetin can modulate Th1/Th2 balance and correct the allergic Th2 drift by synergistic inhibition of IL-4 and upregulating IfN-γ.

Our meta-analysis revealed that quercetin exerts its anti-allergic effects largely through modulating the following critical cytokine pathways: suppression of Th2 cytokines (IL-4, IL-13) and possibly promoting regulatory T cell activity (via FOXP3). The translational significance of modulating these pathways has strong support in human genetics. As shown in the study by Tang et al. (2020), single nucleotide polymorphisms in genes critically involved in immune regulation (IL-18, FOXP3 and IL-13) significantly increase the susceptibility of individuals to allergic rhinitis (Tang et al., 2020). This finding provides a very convincing genetic basis. Dysregulation of the very pathways modulated by quercetin may represent the key mechanism of allergic disease pathogenesis. Therefore, our results support and provide a mechanistic explanation for this genetic phenotype: by suppressing the activity of IL-4/IL-13 signaling pathway and possibly enhancing differentiation of FOXP3+ Treg, quercetin may reverse a critical vulnerability existing in allergic individuals, connecting the preclinical observation with human disease mechanism.

### Exploratory outcomes

Among exploratory outcomes, several biomarkers showed non-significant trends that merit further investigation. Neutrophil counts and IL-5 levels—a cytokine critical for eosinophil maturation and recruitment (Cruz et al., 2008)—displayed downward trends but did not reach statistical significance. Similarly, variations in IL-10 levels, an anti-inflammatory cytokine linked to Treg activity, were inconsistent across studies, with reports of both elevation (Rajizadeh et al., 2023) and suppression (Zhou et al., 2024), suggesting context-dependent modulation.

Notably, quercetin significantly reduced histamine levels, a key biogenic amine in immediate hypersensitivity reactions. Zhou et al. (2024) identified a covalent modification-based mechanism for histamine attenuation, while Shishehbor et al. (2010) observed reduced plasma histamine in clinical allergy models, affirming quercetin’s dual capacity for biochemical and systemic regulation of histaminergic activity.

### Subgroup analysis

By performing subgroup analyses clustered in multiple levels (animal species, disease models, induction methods, administration routes, treatment duration, and sample matrices), we explored the sources of heterogeneity and the consistency of anti-allergic effects of quercetin. Our results suggested that quercetin was usually more effective in mouse models (e.g., significant decrease of IgE, immune cell infiltration, and IL-4 level) than in rats. When stratified by disease models, quercetin’s inhibitory effect on OVA-specific IgE was more evident in allergic rhinitis and asthma models while clear suppression of total IgE was observed in atopic dermatitis models. When stratified by induction methods, improvement of indicators was observed in models induced by different allergens (OVA, DNCB, Df), indicating that quercetin is not limited to suppress allergic reactions against one type of allergen. Subgroup analyses by administration routes showed that both intraperitoneal injection and other administration routes were effective; however, some outcomes showed larger effect sizes with administration through non-intraperitoneal routes (e.g., OVA-IgE). Thus, the administration strategy needs to be further optimized. Subgroup analysis by treatment duration showed that quercetin’s effect on most outcomes was stronger with treatment duration <30 days (e.g., IgE, OVA-IgE, HIS), while treatment for ≥30 days still significantly suppressed total IgE, macrophages, lymphocytes, and eosinophils. In addition, the effect on most outcomes was still significant when the treatment duration was ≥30 days. Furthermore, the effect on different outcomes was also influenced by sample matrices (e.g., serum, BALF, and tissue). For example, the inhibition of IgE and IL-5 in serum and histamine in tissue were most significant. Although significant differences existed in effect estimates among subgroups, querc still significantly improved core allergy-related indicators in different models and species, confirming quercetin’s position as a multi-target active molecule against allergies and providing empirical support for the design of key parameters in subsequent translational research.

The validity of our translational hypothesis depends on the well-established roles of the biomarkers analyzed. The multi-target reductions achieved--from initial IgE sensitization and histamine release to downstream cellular infiltration and cytokine-driven inflammation--represent suppression of the entire allergic cascade. Therefore, the consistent direction and statistical significance of these effects in different models are not some isolated pharmacological effects but strong preclinical evidence of quercetin’s potential therapeutic efficacy. In other words, quercetin shows potential as a compound that suppresses the pathophysiology of allergic diseases instead of a single symptom.

### Nanomedicine strategy: a frontier direction for overcoming the limitations of quercetin ADME

Despite its remarkable anti-allergic activity in animal models, quercetin is limited in clinical application due to its own pharmacokinetic properties, including low water solubility, low intestinal absorption, poor metabolic stability and fast systemic clearance. To overcome these ADME limitations in a systematic way, nanomaterial strategies have gradually attracted attentions. Numerous nanocarriers (including liposomes, polymer nanoparticles, solid lipid nanoparticles and nanocrystals) greatly improve quercetin efficacy at different aspects. Quercetin nanocrystals prepared by high-pressure homogenization could greatly improve its solubility and stability in aqueous solution, and showed better pharmacokinetic behavior and anti-allergic effects compared with ordinary quercetin in rat asthma models. It could significantly decrease the levels of allergic immunoglobulins and inflammatory factors at low dose (Gupta et al., 2016). On the other hand, the above-mentioned advantages of nanocarriers for quercetin are also achieved by enhancing its solubility and dissolution rate by increasing specific surface area, enhancing bioadhesion and endocytosis by intestinal immune cells to improve absorption, and even further improving the lymphatic transport to bypass the first-pass effect. Just as casein nanoparticles can enhance the absorption of quercetin by improving its entero-immune cell endocytosis and facilitating its absorption through intestinal lymphatic transport, which further improve the bioavailability and accumulate in intestinal associated immune tissues (Mazhar et al., 2025).

### Clinical practice and future prospects

Quercetin exhibits anti-therapeutic effects in preclinical models through suppression of IgE, modulation of immune cells, and rebalancing of Th1/Th2 cytokines, indicating its potential for use as an adjunct therapy for allergic diseases. However, translation into clinical practice necessitates well-designed human trials to confirm its efficacy, safety, and optimal dose. Currently, the existence of heterozygous study designs, species-specific responses, and limited information on safety pose challenges to the use of multicenter, randomized controlled trials with standardized protocols. In the future, efforts should be made to investigate the molecular mechanisms of quercetin in humans, understand its potential synergy with existing drugs, and develop bioenhanced quercetin to improve the oral bioavailability. Only then can we bridge the preclinical findings to clinical application, providing a natural multi-target approach for the treatment of allergy.

However, the critical appraisal of quercetin’s translational potential should not stop at its mechanistic efficacy, but should also account for its suboptimal pharmacokinetic profile.

Although our study and other reports have demonstrated the bioactivities of quercetin in cellular and animal models, the translational potential of quercetin should be rigorously evaluated considering its suboptimal pharmacokinetics.

Quercetin exhibits notoriously low oral bioavailability due to its extensive pre-systemic metabolism through glucuronidation, sulfation, and methylation in the intestine and liver (Abu-Amero et al., 2016). Therefore, the systemic exposure of the parent, pharmacologically active aglycone would be minimal and transient. The pharmacokinetic issue renders quercetin in a great paradox: the efficacious dose of quercetin in rodent models often ranges between 50 and 150 mg/kg. However, this high dose of quercetin in animals translates into minimal human dose because of its low bioavailability. When converting the high animal dose into Human Equivalent Doses (HED) by normalizing to human body surface area, the quercetin dose is much higher than that could be achieved by dietary intake or even available commercial quercetin supplements in humans (Chin et al., 2025). Therefore, the efficacy observed in preclinical settings should be calibrated against these pharmacological realities. The positive effect observed in animals would not translate into humans unless special strategies to improve its bioavailability (such as the use of advanced formulations like nanoparticles or co-administration with metabolism inhibitors) are adopted. Therefore, the quercetin field should embrace the need for “PAINS-aware” designs that not only improve target specificity but also solve pharmacologically critical issues of bioavailability and metabolic stability simultaneously to generate truly druggable quercetin-inspired molecules.

In recent years, in order to improve the oral bioavailability of quercetin, a large number of new formulation strategies have been developed and have entered the preclinical and early clinical research stage. For example, the systemic exposure and *in vivo* efficacy of quercetin were greatly improved through enhancing the water solubility, delaying metabolism and enhancing the intestinal absorption by carrier systems such as liposomes, phospholipid complexes (phytosomes), nanocrystals and polymer nanoparticles. The emergence of these formulation technologies provide an effective and feasible solution to overcome the pharmacokinetic disadvantages of quercetin and advance its clinical application.

### Strengths and limitations

This review followed PRISMA guidelines closely with a thorough synthesis of preclinical evidence supporting quercetin’s immunomodulatory actions in allergic diseases.

Strengths include good methodological design, consistency in identification of modulated biomarkers across studies and generation of a translational research framework. However, caution is warranted.

First, the applicability of our results to humans may be impacted by the characteristics of the animal models synthesized. As indicated in the methods, the analysis summarized data from young adult rodents (6–8 weeks old). In addition, although both male and female animals were included, animal primary studies did not provide data stratified by sex, which means that the potential sex-dependent effects of quercetin cannot be assessed and should be considered as an important next step.

Second, the output of this meta-analysis should be interpreted as follows: although all animal primary studies included in this review reported data using individual animal as the unit of analysis and did not explicitly mention cage-level intervention or cluster designs, we did not statistically adjust for potential “cage effects”.

Furthermore, although this meta-analysis was not adjusted for RVE because animal primary studies included in this review did not explicitly use a clustering design, we acknowledge the advance that has been made in this direction. For example, simulation-based small-sample RVE adjustment methods (based on Satterthwaite degrees of freedom approximation or bias correction) have been developed, specifically aimed at statistical inference when number of clusters is limited (e.g., k < 10 or 20) (Pustejovsky and Tipton, 2018; Tipton and Pustejovsky, 2015). In the future, there will be more accurate effect size estimates and standard errors when more sophisticated clustering designs (e.g., randomization at the cage level) are frequently used and reported in primary animal studies.

In addition, this study did not combine data from “paired tissues” (e.g., symmetrical organs) from one individual. Future systematic reviews of animal studies should use more sophisticated statistical methods [e.g., adjust sample sizes (design effects) or fit multi - level models] to deal with non - independence when included studies use explicit cluster designs or report paired data.

Third, quantitative analysis of biomarkers was limited by the small number of eligible studies for further large-scale replication. Furthermore, the heterogeneous chemical forms of quercetin targets (aglycone vs. glycosides) used in studies might lead to the aforementioned heterogeneity; however, the similar trends of effects across different outcomes seem to be robust with regard to the core immunomodulatory activity of quercetin.

Our analysis focused on immunomodulation. However, the protective profile of quercetin extends to novel pathways involved in the pathogenesis of multiple diseases, suggesting other pleiotropic mechanisms apart from its classical immunomodulation and anti-inflammatory activities. For example, it has been reported that quercetin could inhibit hippocampal ferroptosis in diabetic encephalopathy (Cheng et al., 2024), indicating a pleiotropic action of quercetin that might not be reflected by its classical readout (i.e., antioxidant activity).

In addition, the large extent of study heterogeneity and methodological limitations identified in our study are not an isolated incident. In fact, this issue seems to be universal for preclinical meta-analyses of quercetin (or other natural products). For example, in our study on models of acute kidney injury, Zeng et al. also found a significant protective effect of quercetin but explicitly stated that their conclusions were substantially limited by high heterogeneity and risk of bias in the primary studies (Zeng et al., 2023). Therefore, the parallel findings of quercetin protection in different organ systems indicate that addressing the core issues of experimental design and reporting standardization should be given priority in future research to improve the reliability and translational relevance of preclinical evidence for quercetin and other natural products.

Furthermore, the PAINS (pan-assay interference compounds) nature of quercetin aglycone should be considered. The potential to mediate redox cycling, metal chelation, and nonspecific binding may lead to false-positive readouts in certain assay systems (Adasme et al., 2021). Although our review includes mostly *in vivo* studies, which are less prone to these artifacts, the possibility of confounding effects in underlying mechanistic studies cannot be completely ruled out. Future research should use PAINS-aware experimental designs (including the use of counterassays and metabolite verification) to ensure result validity.

Compared with the previous method, the new meta-analysis showed similar directions of effects for most outcomes, while the SMDs were generally decreased and CIs narrowed with more changes in heterogeneity. For example, the SMD of total IgE was changed from −4.28 (95% CI: −6.07, −2.48; I^2^ = 87%) to −3.59 (95% CI: −5.17, −2.01; I^2^ = 89%). The results of OVA-IgE, macrophages, lymphocytes, eosinophils, IL-4, TNF-α, IFN-γ, and histamine were also similar, while effect sizes decreased but the significance remained (all p < 0.05). The effect of IL-5 changed from borderline significant to significantly negative (p < 0.00001) and the heterogeneity decreased (I^2^ = 49%) (Figure 9). These results indicated that quercetin has suppressed IgE and modulated Th1/Th2, and these core effects were not influenced by the selection of quercetin concentration.

In the new quantitative synthesis, we conducted sensitivity analysis by pooling data from multiple quercetin concentrations (as in Park et al. (2009), Ding et al. (2020), Chen and Ko (2021)) instead of using only the highest concentration. In addition, although Egger’s and Begg’s tests were used to examine whether there was potential publication bias on multiple outcomes, including IgE, OVA-IgE, cytokines (IL-4, IL-5, IL-10, TNF-α, IFN-γ), immune cells (macrophages [Mac], lymphocytes [Lym], eosinophils [Eos], neutrophils [Neu]) and histamine, several core indicators of allergy, such as IgE, EOS, IL-4 and IL-5, showed significant publication bias, which may influence the accuracy of effect size estimation. However, most indicators (such as OVA-IgE, TNF-α and histamine) showed robust results. Although the sensitivity analysis proved that the overall risk of bias of pooled effect estimates was moderate, the combined existence of high heterogeneity, wide confidence intervals and common methodological flaws for included studies led to a moderate overall risk of bias rating. That is, we have moderate confidence in the effect estimates and acknowledge that the true effect may differ, but the effect estimates based on the included studies consistently indicated that quercetin can significantly decrease the levels of IgE and OVA-IgE.

Furthermore, another important source of bias was found related to the quality of the included studies (i.e., the methodological quality assessed by the included studies) as assessed by SYRCLE’s risk of bias assessment.

The majority of judgments regarding the risk of bias were “Unclear” or “High” for several aspects of methodology, such as sequence generation, allocation concealment, and blinding of personnel. According to the methodological research established for Cochrane reviews, these biases are likely to result in an inflation of the effect estimate in a direction (Hooijmans et al., 2014). For instance, if sequence generation and allocation concealment are inadequate, selection bias will be introduced, and subjects with better prognoses may be systematically assigned to the intervention group. This would lead to an overestimation of the true treatment effect. If personnel are not blinded to allocation, either by the care provider or the outcome assessor (especially for subjective outcomes), performance and detection bias will also be introduced, which likewise typically leads to an overestimation of benefit. Considering the overall trend towards a positive effect was observed in our meta-analysis, the presence of these potential biases may slightly inflate the magnitude of the pooled effect size. Therefore, the beneficial effects of quercetin that have been interpreted in this study should be interpreted with caution. Studies using more robust methods for sequence generation, allocation concealment, and blinding will be necessary to provide more unbiased and accurate estimates of the effect of the intervention.

Finally, translational relevance is unknown because of the inherent differences between the animal model and human allergic disease pathophysiology. The therapeutic potential of quercetin as an adjuvant has been demonstrated in this study; however, the clinical application remains to be demonstrated in standardized multicenter human trials.

In summary, our findings demonstrate potential for flavonoid research and gaps in the current evidence.

Interestingly, our findings on the immunomodulatory effects of quercetin share a similar theme with the pharmacology of other natural products, such as Astragalus. Parallel to our findings on quercetin, Astragalus has also been demonstrated to modulate intestinal microbiota to improve barrier function and immunity in metabolic disease (Su et al., 2023). This line of evidence supports the notion that phytochemicals are multi-target immunoregulators that modulate host immunity through both direct signaling and indirect, microbiota-dependent mechanisms.

## Conclusion

In summary, this meta-analysis showed that quercetin possesses robust anti-allergic activities in preclinical models through a multi-tier immunomodulatory action stratified according to pre-defined outcome hierarchies. Quercetin consistently attenuated underlying allergic pathology in primary targets by significantly down-regulating total IgE production, eosinophilic infiltration, and levels of the key Th2 cytokine IL-4. These endpoints represent fundamental characteristics of allergic inflammation, suggesting quercetin exerts effects on primary disease pathologies. Quercetin modulated a broader range of immunological processes beyond primary targets. Quercetin significantly inhibited OVA-specific IgE, showed dose-dependent significant reductions in macrophage and lymphocyte counts, rebalanced the Th1/Th2 axis by down-regulating TNF-α and up-regulating IFN-γ, and further downregulated IL-4, enhancing the shift away from Th2 polarization. In exploratory analyses, quercetin significantly downregulated histamine, the prototypical mediator of immediate acute allergic symptoms, providing further support for the clinical phenotypes of subjective allergic symptoms. Other endpoints such as IL-5, IL-10, and neutrophils showed suggestive effects but merit further investigation.

Interestingly, a recent open-label clinical trial in children with allergic rhinitis (Gori et al., 2025) yielded preliminary human evidence that a multi-component supplement containing quercetin significantly improved objective biomarkers of allergy. However, it is important to note that the open-label study involved a multi-ingredient formulation and that the effects may be due to synergistic interactions among multiple components rather than quercetin *per se*. Additionally, the study design precluded a placebo control and limited the scope of findings; thus, these promising findings should be confirmed by large-scale, RCTs designs. These convergent findings support the potential translational application of multi-component formulations containing quercetin. However, we must emphasize that there is currently no strong RCT evidence to support the clinical use of a single-ingredient quercetin as a standard medical treatment for allergic rhinitis. The large heterogeneity in the preclinical evidence base also limits interpretation but highlights the need for rigorous human trials to evaluate efficacy, safety, and optimal dosing.

## Data Availability

The datasets presented in this study can be found in online repositories. The names of the repository/repositories and accession number(s) can be found in the article/Supplementary Material.

## References

[B1] Abu-Amero K. K. Kondkar A. A. Chalam K. V. (2016). Resveratrol and ophthalmic diseases. Nutrients 8 (4), 200. 10.3390/nu8040200 27058553 PMC4848669

[B68] Adasme M. F. Linnemann K. L. Bolz S. N. Kaiser F. Salentin S. Haupt V. J. (2021). PLIP 2021: expanding the scope of the protein-ligand interaction profiler to DNA and RNA. Nucleic Acids Res. 49, W530–W534. 10.1093/nar/gkab294 33950214 PMC8262720

[B2] Agache I. Akdis C. A. (2019). Precision medicine and phenotypes, endotypes, genotypes, regiotypes, and theratypes of allergic diseases. J. Clin. Invest. 129 (4), 1493–1503. 10.1172/jci124611 30855278 PMC6436902

[B3] Agache I. Miller R. Gern J. E. Hellings P. W. Jutel M. Muraro A. (2019). Emerging concepts and challenges in implementing the exposome paradigm in allergic diseases and asthma: a practall document. Allergy 74 (3), 449–463. 10.1111/all.13690 30515837

[B4] Akdis C. A. (2012). Therapies for allergic inflammation: refining strategies to induce tolerance. Nat. Med. 18 (5), 736–749. 10.1038/nm.2754 22561837

[B5] Atanasio A. Orengo J. M. Sleeman M. A. Stahl N. (2022). Biologics as novel therapeutics for the treatment of allergy: challenges and opportunities. Front. Allergy 3, 1019255. 10.3389/falgy.2022.1019255 36353195 PMC9637826

[B6] Atanasov A. G. Zotchev S. B. Dirsch V. M. Supuran C. T. (2021). Natural products in drug discovery: advances and opportunities. Nat. Rev. Drug Discov. 20 (3), 200–216. 10.1038/s41573-020-00114-z 33510482 PMC7841765

[B7] Aw Yong P. Y. Islam F. Harith H. H. Israf D. A. Tan J. W. Tham C. L. (2020). The potential use of honey as a remedy for allergic diseases: a mini review. Front. Pharmacol. 11, 599080. 10.3389/fphar.2020.599080 33574752 PMC7870997

[B8] Bernini R. Velotti F. (2021). Natural polyphenols as immunomodulators to rescue immune response homeostasis: Quercetin as a research model against severe COVID-19. Molecules 26 (19), 5803. 10.3390/molecules26195803 34641348 PMC8510228

[B9] Bieber T. (2022). Atopic dermatitis: an expanding therapeutic pipeline for a complex disease. Nat. Rev. Drug Discov. 21 (1), 21–40. 10.1038/s41573-021-00266-6 34417579 PMC8377708

[B10] Bousquet J. Grattan C. E. Akdis C. A. Eigenmann P. A. Hoffmann-Sommergruber K. Agache I. (2020a). Highlights and recent developments in allergic diseases in EAACI journals (2019). Clin. Transl. Allergy 10 (1), 56. 10.1186/s13601-020-00366-3 33292572 PMC7712618

[B11] Bousquet J. Schünemann H. J. Togias A. Bachert C. Erhola M. Hellings P. W. (2020b). Next-generation allergic rhinitis and its impact on asthma (ARIA) guidelines for allergic rhinitis based on grading of recommendations assessment, development and evaluation (GRADE) and real-world evidence. J. Allergy Clin. Immunol. 145 (1), 70–80.e3. 10.1016/j.jaci.2019.06.049 31627910

[B12] Cai Y. Wang L. Zhang X. Liu H. (2017). Quercetin inhibits airway allergic inflammation through inhibiting the expression of NF-κB activator 1. Int. J. Clin. Exp. Med. 10 (6), 9238–9244. Available online at: http://www.ijcem.com /ISSN:1940-5901/IJCEM0047309.

[B69] Chen J. Lin Z. Ding J. Zusanli (ST36) (2021). Acupoint injection with dexamethasone for chemotherapy-induced myelosuppression: a systematic review and meta-analysis. Front. Oncol. 11, 684129. 10.3389/fonc.2021.684129 34295820 PMC8291031

[B13] Chen L. W. Ko W. C. (2021). Suppressive effects of rutin, quercitrin, and isoquercitrin on atypical allergic asthma in an animal model. Med. Drug Discov. 12, 100106. 10.1016/j.medidd.2021.100106

[B14] Cheng X. Huang J. Li H. Zhao D. Liu Z. Zhu L. (2024). Quercetin: a promising therapy for diabetic encephalopathy through inhibition of hippocampal ferroptosis. Phytomedicine 126, 154887. 10.1016/j.phymed.2023.154887 38377720

[B15] Chiang M. C. Tsai T. Y. Wang C. J. (2023). The potential benefits of quercetin for brain health: a review of anti-inflammatory and neuroprotective mechanisms. Int. J. Mol. Sci. 24 (7), 6328. 10.3390/ijms24076328 37047299 PMC10094159

[B16] Chin S. P. Abd Rahim E. N. A. Nor Arfuzir N. N. (2025). Neuroprotective effects of human umbilical cord mesenchymal stem cells (Neuroncell-EX) in a rat model of ischemic stroke are mediated by immunomodulation, blood-brain barrier integrity, angiogenesis, and neurogenesis. In Vitro Cell Dev Biol Anim 61 (4), 389–402. 10.1007/s11626-025-01037-y 40360812 PMC12125091

[B17] Cruz E. A. Da-Silva S. A. Muzitano M. F. Silva P. M. Costa S. S. Rossi-Bergmann B. (2008). Immunomodulatory pretreatment with Kalanchoe pinnata extract and its quercitrin flavonoid effectively protects mice against fatal anaphylactic shock. Int. Immunopharmacol. 8 (12), 1616–1621. 10.1016/j.intimp.2008.07.006 18675940

[B18] Cruz E. A. Reuter S. Martin H. Dehzad N. Muzitano M. F. Costa S. S. (2012). Kalanchoe pinnata inhibits mast cell activation and prevents allergic airway disease. Phytomedicine 19 (2), 115–121. 10.1016/j.phymed.2011.06.030 21802918

[B19] Deepika D. Maurya P. K. (2022). Health benefits of quercetin in age-related diseases. Molecules 27 (8), 2498. 10.3390/molecules27082498 35458696 PMC9032170

[B20] Deng Q. Li X. X. Fang Y. Chen X. Xue J. (2020). Therapeutic potential of quercetin as an antiatherosclerotic agent in atherosclerotic cardiovascular disease: a review. Evid. Based Complement. Altern. Med. 2020, 5926381. 10.1155/2020/5926381 32565865 PMC7292974

[B21] Di Lorenzo G. Di Bona D. Belluzzo F. Macchia L. (2017). Immunological and non-immunological mechanisms of allergic diseases in the elderly: biological and clinical characteristics. Immun. Ageing 14, 23. 10.1186/s12979-017-0105-4 29296117 PMC5738884

[B22] Ding Y. Li C. Zhang Y. Ma P. Zhao T. Che D. (2020). Quercetin as a Lyn kinase inhibitor inhibits IgE-mediated allergic conjunctivitis. Food Chem. Toxicol. 135, 110924. 10.1016/j.fct.2019.110924 31672514

[B23] Docampo-Palacios M. L. Alvarez-Hernández A. Adiji O. Gamiotea-Turro D. Valerino-Diaz A. B. Viegas L. P. (2020). Glucuronidation of methylated Quercetin derivatives: Chemical and biochemical approaches. J. Agric. Food Chem. 68 (50), 14790–14807. 10.1021/acs.jafc.0c04500 33289379 PMC8136248

[B24] Dong W. L. An J. Yu M. Yin P. Xu T. L. Liu B. (2021). The prevalence and year lived with disability of atopic dermatitis in China: findings from the global burden of disease study 2019. World Allergy Organ J. 14 (11), 100604. 10.1016/j.waojou.2021.100604 34820052 PMC8591460

[B25] Gori A. Brindisi G. Anania C. Spalice A. Zicari A. M. (2025). Synergic efficacy of a multicomponent nutraceutical Add-On therapy in seasonal allergic rhinitis in children: a prospective, randomized, parallel-group study. J. Clin. Med. 14 (5), 1517. 10.3390/jcm14051517 40094983 PMC11900512

[B26] Gupta K. Kumar S. Gupta R. K. Sharma A. Verma A. K. Stalin K. (2016). Reversion of asthmatic complications and mast cell signalling pathways in BALB/c mice model using Quercetin nanocrystals. J. Biomed. Nanotechnol. 12 (4), 717–731. 10.1166/jbn.2016.2197 27301198

[B27] Hooijmans C. R. Rovers M. M. de Vries R. B. Leenaars M. Ritskes-Hoitinga M. Langendam M. W. (2014). SYRCLE’s risk of bias tool for animal studies. BMC Med. Res. Methodol. 14, 43. 10.1186/1471-2288-14-43 24667063 PMC4230647

[B28] Hossenbaccus L. Linton S. Garvey S. Ellis A. K. (2020). Towards definitive management of allergic rhinitis: best use of new and established therapies. Allergy Asthma Clin. Immunol. 16, 39. 10.1186/s13223-020-00436-y 32508939 PMC7251701

[B70] Hozo S. P. Djulbegovic B. Hozo I. (2005). Estimating the mean and variance from the median, range, and the size of a sample. BMC Med. Res. Methodol. 5, 13. 10.1186/1471-2288-5-13 15840177 PMC1097734

[B29] Jegal J. Park N. J. Lee S. Y. Jo B. G. Bong S. K. Kim S. N. (2020). Quercitrin, the main compound in Wikstroemia indica, mitigates skin lesions in a mouse model of 2,4-Dinitrochlorobenzene-Induced contact hypersensitivity. Evid. Based Complement. Altern. Med. 2020, 4307161. 10.1155/2020/4307161 32695208 PMC7368186

[B30] Kattan J. D. Wang J. (2013). Allergen component testing for food allergy: ready for prime time? Curr. Allergy Asthma Rep. 13 (1), 58–63. 10.1007/s11882-012-0311-2 23011598 PMC4276333

[B31] Kim D. H. Khan H. Ullah H. Hassan S. T. S. Šmejkal K. Efferth T. (2019). MicroRNA targeting by quercetin in cancer treatment and chemoprotection. Pharmacol. Res. 147, 104346. 10.1016/j.phrs.2019.104346 31295570

[B32] Kumar B. Deshmukh R. (2024). A review on novel therapeutic modalities and evidence-based drug treatments against allergic rhinitis. Curr. Pharm. Des. 30 (12), 887–901. 10.2174/0113816128295952240306072100 38486383

[B33] Lambrecht B. N. Hammad H. (2015). The immunology of asthma. Nat. Immunol. 16 (1), 45–56. 10.1038/ni.3049 25521684

[B34] Li Y. Yao J. Han C. Yang J. Chaudhry M. T. Wang S. (2016). Quercetin, inflammation and immunity. Nutrients 8 (3), 167. 10.3390/nu8030167 26999194 PMC4808895

[B35] Linton S. Hossenbaccus L. Ellis A. K. (2023). Evidence-based use of antihistamines for treatment of allergic conditions. Ann. Allergy Asthma Immunol. 131 (4), 412–420. 10.1016/j.anai.2023.07.019 37517656

[B36] Lloyd-Lavery A. Solman L. Grindlay D. J. C. Rogers N. K. Thomas K. S. Harman K. E. (2019). What's new in atopic eczema? An analysis of systematic reviews published in 2016. Part 2: epidemiology, aetiology and risk factors. Clin. Exp. Dermatol 44 (4), 370–375. 10.1111/ced.13853 30706503

[B37] Manach C. Scalbert A. Morand C. Rémésy C. Jiménez L. (2004). Polyphenols: food sources and bioavailability. Am. J. Clin. Nutr. 79 (5), 727–747. 10.1093/ajcn/79.5.727 15113710

[B38] Marquardt D. L. Wasserman S. I. (1982). Mast cells in allergic diseases and mastocytosis. West J. Med. 137 (3), 195–212. 6293204 PMC1274065

[B39] Mazhar A. Hameed A. Ming C. Bhatti S. A. Amir M. Khan M. U. (2025). Development of tolerogenic casein encapsulated quercetin and curcumin nanoparticles to mitigate cow milk allergic responses. Int. J. Biol. Macromol. 314, 144396. 10.1016/j.ijbiomac.2025.144396 40398766

[B40] Meghji J. Mortimer K. Jayasooriya S. Marks G. B. (2021). Lung health in LMICs: tackling challenges ahead - authors’ reply. Lancet 398 (10299), 490. 10.1016/s0140-6736(21)01252-6 34364524

[B41] Mu D. Zhou L. Shi L. Liu T. Guo Y. Chen H. (2024). Quercetin-crosslinked chitosan nanoparticles: a potential treatment for allergic rhinitis. Sci. Rep. 14 (1), 4021. 10.1038/s41598-024-54501-2 38369554 PMC10874938

[B42] Mullowney M. W. Duncan K. R. Elsayed S. S. Garg N. van der Hooft J. J. J. Martin N. I. (2023). Artificial intelligence for natural product drug discovery. Nat. Rev. Drug Discov. 22 (11), 895–916. 10.1038/s41573-023-00774-7 37697042 PMC13118512

[B43] Nasim N. Sandeep I. S. Mohanty S. (2022). Plant-derived natural products for drug discovery: current approaches and prospects. Nucl. (Calcutta) 65 (3), 399–411. 10.1007/s13237-022-00405-3 36276225 PMC9579558

[B44] Nemeth K. Piskula M. K. (2007). Food content, processing, absorption and metabolism of onion flavonoids. Crit. Rev. Food Sci. Nutr. 47 (4), 397–409. 10.1080/10408390600846291 17457724

[B45] Nguyen T. L. A. Bhattacharya D. (2022). Antimicrobial activity of quercetin: an approach to its mechanistic principle. Molecules 27 (8), 2494. 10.3390/molecules27082494 35458691 PMC9029217

[B46] Ogulur I. Pat Y. Ardicli O. Barletta E. Cevhertas L. Fernandez-Santamaria R. (2021). Advances and highlights in biomarkers of allergic diseases. Allergy 76 (12), 3659–3686. 10.1111/all.15089 34519063 PMC9292545

[B47] Park H. J. Lee C. M. Jung I. D. Lee J. S. Jeong Y. I. Chang J. H. (2009). Quercetin regulates Th1/Th2 balance in a murine model of asthma. Int. Immunopharmacol. 9 (3), 261–267. 10.1016/j.intimp.2008.10.021 19061976

[B48] Park E. J. Kim J. Y. Jeong M. S. Park K. Y. Park K. H. Lee M. W. (2015). Effect of topical application of quercetin-3-O-(2″-gallate)-α-l-rhamnopyranoside on atopic dermatitis in NC/Nga mice. J. Dermatol Sci. 77 (3), 166–172. 10.1016/j.jdermsci.2014.12.005 25617237

[B49] Pawankar R. (2014). Allergic diseases and asthma: a global public health concern and a call to action. World Allergy Organ J. 7 (1), 12. 10.1186/1939-4551-7-12 24940476 PMC4045871

[B50] Pustejovsky J. E. Tipton E. (2018). Small-sample methods for cluster-robust variance estimation and hypothesis testing in fixed effects models. J. Bus. and Econ. Statistics 36 (4), 672–683. 10.1080/07350015.2016.1247004

[B51] Qian L. Mehrabi Nasab E. Athari S. M. Athari S. S. (2022). Mitochondria signaling pathways in allergic asthma. J. Investig. Med. 70 (4), 863–882. 10.1136/jim-2021-002098 35168999 PMC9016245

[B52] Rajizadeh M. A. Bejeshk M. A. Doustimotlagh A. H. Najafipour H. Eftekhari M. Mahmoodi M. (2023). The alleviating impacts of Quercetin on inflammation and oxidant-antioxidant imbalance in rats with allergic asthma. Iran. J. Allergy Asthma Immunol. 22 (2), 138–149. 10.18502/ijaai.v22i2.12675 37496407

[B53] Ren L. Yan L. Shi W. Zhang T. Geng B. Mao J. (2023). Evaluation of subchronic toxicity of the compound of diphenhydramine hydrochloride and caffeine after 28 days of repeated oral administration in Sprague-Dawley rats and beagle dogs. Drug Chem. Toxicol. 46 (6), 1083–1099. 10.1080/01480545.2022.2129674 36384384

[B54] Sagit M. Polat H. Gurgen S. G. Berk E. Guler S. Yasar M. (2017). Effectiveness of quercetin in an experimental rat model of allergic rhinitis. Eur. Arch. Otorhinolaryngol. 274 (8), 3087–3095. 10.1007/s00405-017-4602-z 28493194

[B55] Shishehbor F. Behroo L. Ghafouriyan Broujerdnia M. Namjoyan F. Latifi S. M. (2010). Quercetin effectively quells peanut-induced anaphylactic reactions in the peanut sensitized rats. Iran. J. Allergy Asthma Immunol. 9 (1), 27–34. 20548131

[B56] Sitek A. Chiarella S. E. Pongdee T. (2023). Hypersensitivity reactions to biologics used in the treatment of allergic diseases: clinical features, diagnosis and management. Front. Allergy 4, 1219735. 10.3389/falgy.2023.1219735 37637139 PMC10450930

[B57] Sterne J. A. Sutton A. J. Ioannidis J. P. Terrin N. Jones D. R. Lau J. (2011). Recommendations for examining and interpreting funnel plot asymmetry in meta-analyses of randomised controlled trials. Bmj 343, d4002. 10.1136/bmj.d4002 21784880

[B58] Su M. Tang T. Tang W. Long Y. Wang L. Liu M. (2023). Astragalus improves intestinal barrier function and immunity by acting on intestinal microbiota to treat T2DM: a research review. Front. Immunol. 14, 1243834. 10.3389/fimmu.2023.1243834 37638043 PMC10450032

[B59] Tang L. Chen Y. Xiang Q. Xiang J. Tang Y. Li J. (2020). The association between IL18, FOXP3 and IL13 genes polymorphisms and risk of allergic rhinitis: a meta-analysis. Inflamm. Res. 69 (9), 911–923. 10.1007/s00011-020-01368-4 32529476

[B60] Tipton E. Pustejovsky J. E. (2015). Small-sample adjustments for tests of moderators and model fit using robust variance estimation in meta-regression. J. Educ. Behav. Statistics 40 (6), 604–634. 10.3102/1076998615606099

[B61] Tsoi L. C. Rodriguez E. Stölzl D. Wehkamp U. Sun J. Gerdes S. (2020). Progression of acute-to-chronic atopic dermatitis is associated with quantitative rather than qualitative changes in cytokine responses. J. Allergy Clin. Immunol. 145 (5), 1406–1415. 10.1016/j.jaci.2019.11.047 31891686 PMC7214216

[B62] Wang J. Zhou Y. Zhang H. Hu L. Liu J. Wang L. (2023). Pathogenesis of allergic diseases and implications for therapeutic interventions. Signal Transduct. Target Ther. 8 (1), 138. 10.1038/s41392-023-01344-4 36964157 PMC10039055

[B63] Warren C. M. Turner P. J. Chinthrajah R. S. Gupta R. S. (2021). Advancing food allergy through epidemiology: understanding and addressing disparities in food allergy management and outcomes. J. Allergy Clin. Immunol. Pract. 9 (1), 110–118. 10.1016/j.jaip.2020.09.064 33065370 PMC7938932

[B64] Xiao L. Gong H. Tan X. Chen P. Yang Y. Zhu H. (2025). Physicochemical characterization and antitumor activity *in vitro* of a polysaccharide from Christia vespertilionis. Int. J. Biol. Macromol. 290, 139095. 10.1016/j.ijbiomac.2024.139095 39722381

[B65] Yao Y. Chen C. L. Yu D. Liu Z. (2021). Roles of follicular helper and regulatory T cells in allergic diseases and allergen immunotherapy. Allergy 76 (2), 456–470. 10.1111/all.14639 33098663

[B66] Zeng Y. F. Li J. Y. Wei X. Y. Ma S. Q. Wang Q. G. Qi Z. (2023). Preclinical evidence of reno-protective effect of quercetin on acute kidney injury: a meta-analysis of animal studies. Front. Pharmacol. 14, 1310023. 10.3389/fphar.2023.1310023 38186644 PMC10770850

[B67] Zhou E. Li Q. Xu R. Pan F. Tao Y. Li X. (2024). Covalent conjugation with Quercetin mitigates allergenicity of the bee pollen allergen Bra c p in a murine model. Food Chem. 436, 137722. 10.1016/j.foodchem.2023.137722 37857207

