# Supplementary material for: Quercetin exhibits multi-target anti-allergic effects in animal models: a systematic review and meta-analysis of preclinical studies
Source: Front Pharmacol. 2025 Nov 20;16:1673712. doi: 10.3389/fphar.2025.1673712 (PMC12676024; doi:10.3389/fphar.2025.1673712)
Supplement: Supplementary file 1 [file Table1.docx]

**Table 1.** Search strategy

| **Databases** | **Search expression** | **Quantity** |
| --- | --- | --- |
| Pubmed | (quercetin) AND ((((((((allergic rhinitis) OR (Allergic conjunctivitis)) OR (Allergic asthma)) OR (Henoch-Schonlein purpura)) OR (Atopic dermatitis)) OR (urticaria)) OR (Anaphylactic shock)) OR (allergic)) | 260 |
| Web of Science | (quercetin) AND ((((((((allergic rhinitis) OR (Allergic conjunctivitis)) OR (Allergic asthma)) OR (Henoch-Schonlein purpura)) OR (Atopic dermatitis)) OR (urticaria)) OR (Anaphylactic shock)) OR (allergic)) | 245 |
| Embase | ('quercetin'/exp OR quercetin) AND ('allergic rhinitis'/exp OR 'allergic rhinitis' OR (allergic AND ('rhinitis'/exp OR rhinitis)) OR 'allergic conjunctivitis'/exp OR 'allergic conjunctivitis' OR (allergic AND ('conjunctivitis'/exp OR conjunctivitis)) OR 'allergic asthma'/exp OR 'allergic asthma' OR (allergic AND ('asthma'/exp OR asthma)) OR 'henoch-schonlein purpura'/exp OR 'henoch-schonlein purpura' OR ('henoch schonlein' AND ('purpura'/exp OR purpura)) OR 'atopic dermatitis'/exp OR 'atopic dermatitis' OR (atopic AND ('dermatitis'/exp OR dermatitis)) OR 'urticaria'/exp OR urticaria OR 'anaphylactic shock'/exp OR 'anaphylactic shock' OR (anaphylactic AND ('shock'/exp OR shock)) OR allergic) | 715 |

Time:2025-04-17
