# Supplementary material for: Quercetin exhibits multi-target anti-allergic effects in animal models: a systematic review and meta-analysis of preclinical studies
Source: Front Pharmacol. 2025 Nov 20;16:1673712. doi: 10.3389/fphar.2025.1673712 (PMC12676024; doi:10.3389/fphar.2025.1673712)
Supplement: Supplementary file 2 [file Table8.docx]

**Table 6.**Subgroup analysis by sample matrix

| **Outcome** | **Subgroup** | **n(k)** | **N** | **I^2^** | **P(het)** | **SMD** | **95%CI** | **P(effect)** | **P(between)** |
| --- | --- | --- | --- | --- | --- | --- | --- | --- | --- |
| IgE | Serum | 8 | 64 | 87% | <0.001 | -4.28 | [-6.07,-2.48] | <0.001 | - |
|  | - | 0 | 0 | - | - | - | - | - |  |
|  |  |  |  |  |  |  |  |  |  |
| OVA-IgE | Serum | 5 | 38 | 78% | 0.001 | -3.73 | [-5.66,-1.81] | <0.001 | - |
|  | - | 0 | 0 | - | - | - | - | - |  |
|  |  |  |  |  |  |  |  |  |  |
| Mac | Serum | 1 | 5 | - | - | -3.84 | [-6.33,-1.35] | <0.001 | 0.33 |
|  | BALF | 3 | 24 | 87% | <0.001 | -2.19 | [-4.39,0.02] | 0.05 |  |
|  |  |  |  |  |  |  |  |  |  |
| Lym | Serum | 1 | 5 | - | - | -4.68 | [-7.59,-1.77] | <0.001 | 0.36 |
|  | BALF | 3 | 24 | 87% | <0.001 | -2.88 | [-5.40,-0.36] | 0.03 |  |
|  |  |  |  |  |  |  |  |  |  |
| Neu | Serum | 1 | 5 | - | - | -3.14 | [-5.31,-0.98] | <0.001 | 0.42 |
|  | BALF | 3 | 24 | 93% | <0.001 | -1.62 | [-4.64,1.41] | 0.29 |  |
|  |  |  |  |  |  |  |  |  |  |
| Eos | Serum | 2 | 9 | 0% | 0.41 | -8.24 | [-12.01,-4.47] | <0.001 | 0.08 |
|  | Tissue | 2 | 17 | 75% | 0.04 | -4.8 | [-9.38,-0.22] | 0.04 |  |
|  | BALF | 3 | 24 | 82% | <0.001 | -3.17 | [-5.43,-0.91] | <0.001 |  |
|  |  |  |  |  |  |  |  |  |  |
| IL-4 | Serum | 3 | 24 | 92% | <0.001 | -8.25 | [-14.82,-1.67] | 0.01 | 0.33 |
|  | Tissue | 1 | 8 | - | - | -4.12 | [-6.03,-2.20] | <0.001 |  |
|  | BALF | 3 | 21 | 91% | <0.001 | -2.52 | [-6.33,1.29] | 0.19 |  |
|  |  |  |  |  |  |  |  |  |  |
| IL-5 | Serum | 2 | 13 | 0% | 0.49 | -9.71 | [-12.98,-6.44] | <0.001 | 0.05 |
|  | Tissue | 1 | 4 | - | - | -4.26 | [-7.50,-1.02] | 0.01 |  |
|  | BALF | 2 | 15 | 94% | <0.001 | -2.83 | [-13.91,8.25] | 0.62 |  |
|  |  |  |  |  |  |  |  |  |  |
| IL-10 | Tissue | 3 | 19 | 91% | <0.001 | 1.65 | [-3.38,6.69] | 0.52 | - |
|  | - | 0 | 0 | - | - | - | - | - |  |
|  |  |  |  |  |  |  |  |  |  |
| TNF-α | Serum | 2 | 20 | 0% | 0.81 | -6.26 | [-7.92,-4.61] | <0.001 | 0.002 |
|  | Tissue | 2 | 11 | 82% | 0.02 | -5.59 | [-14.80,3.62] | 0.23 |  |
|  | BALF | 2 | 15 | 93% | <0.001 | 0.57 | [-2.77,3.91] | 0.74 |  |
|  |  |  |  |  |  |  |  |  |  |
| IFN-γ | Serum | 2 | 13 | 90% | 0.001 | 4.56 | [-4.53,13.65] | 0.33 | 0.7 |
|  | BALF | 2 | 16 | 94% | <0.001 | 2.55 | [-2.20,7.31] | 0.29 |  |
|  |  |  |  |  |  |  |  |  |  |
| HIS | Serum | 1 | 7 | - | - | -1.72 | [-3.01,-0.43] | 0.009 | <0.001 |
|  | Tissue | 2 | 20 | 0% | 0.49 | -5.74 | [-7.29,-4.20] | <0.001 |  |

n (k) = number of studies; N = total number of animals.
