# Supplementary material for: Quercetin exhibits multi-target anti-allergic effects in animal models: a systematic review and meta-analysis of preclinical studies
Source: Front Pharmacol. 2025 Nov 20;16:1673712. doi: 10.3389/fphar.2025.1673712 (PMC12676024; doi:10.3389/fphar.2025.1673712)
Supplement: Supplementary file 3 [file Table12.docx]

**Table 9.**Publication bias

|  |  | **Begg's Test** | | **Egger's test** | |
| --- | --- | --- | --- | --- | --- |
| Indicator | n | Z | P | Z | P |
| IgE | 8 | -2.23 | 0.026 | -4.81 | 0.003 |
| OVA-IgE | 5 | -1.47 | 0.142 | -2.85 | 0.065 |
| Mac | 4 | -1.36 | 0.174 | -1.65 | 0.241 |
| Lym | 4 | -0.68 | 0.497 | -2.11 | 0.169 |
| Neu | 4 | -1.36 | 0.174 | -3.73 | 0.065 |
| Eos | 7 | -2.25 | 0.024 | -3.44 | 0.018 |
| IL-4 | 7 | -1.95 | 0.051 | -7.84 | 0.001 |
| IL-5 | 5 | -1.47 | 0.142 | -9.72 | 0.002 |
| IL-10 | 3 | -1.57 | 0.117 | -5.29 | 0.119 |
| TNF-α | 6 | -1.32 | 0.188 | -2.14 | 0.099 |
| IFN-γ | 4 | 1.36 | 0.174 | 2.66 | 0.117 |
| HIS | 3 | -0.52 | 0.602 | -2.26 | 0.266 |
