# Supplementary material for: Quercetin exhibits multi-target anti-allergic effects in animal models: a systematic review and meta-analysis of preclinical studies
Source: Front Pharmacol. 2025 Nov 20;16:1673712. doi: 10.3389/fphar.2025.1673712 (PMC12676024; doi:10.3389/fphar.2025.1673712)
Supplement: Supplementary file 4 [file Table7.docx]

**Table 5.**Subgroup analysis by induction model

| **Outcome** | **Subgroup** | **n(k)** | **N** | **I^2^** | **P(het)** | **SMD** | **95%CI** | **P(effect)** | **P(between)** |
| --- | --- | --- | --- | --- | --- | --- | --- | --- | --- |
| IgE | OVA-induced | 5 | 45 | 91% | <0.001 | -5.7 | [-8.67,-2.72] | <0.001 | 0.01 |
|  | DNCB-induced | 1 | 7 | - | - | -2.65 | [-4.21,-1.09] | <0.001 |  |
|  | Df-induced | 1 | 5 | - | - | -6 | [-9.60,2.40] | 0.001 |  |
|  | CPE-induced | 1 | 7 | - | - | -1.44 | [-2.66,-0.22] | 0.02 |  |
|  |  |  |  |  |  |  |  |  |  |
| OVA-IgE | OVA-induced | 5 | 38 | 78% | 0.001 | -3.73 | [-5.66,-1.81] | <0.001 | - |
|  | - | 0 | 0 | - | - | - | - | - |  |
|  |  |  |  |  |  |  |  |  |  |
| Mac | OVA-induced | 4 | 29 | 83% | <0.001 | -2.52 | [-4.43,-0.61] | 0.01 | - |
|  | - | 0 | 0 | - | - | - | - | - |  |
|  |  |  |  |  |  |  |  |  |  |
| Lym | OVA-induced | 4 | 29 | 83% | <0.001 | -3.24 | [-5.43,-1.04] | <0.001 | - |
|  | - | 0 | 0 | - | - | - | - | - |  |
|  |  |  |  |  |  |  |  |  |  |
| Neu | OVA-induced | 4 | 29 | 91% | <0.001 | -1.96 | [-4.53,0.60] | 0.13 | - |
|  | - | 0 | 0 | - | - | - | - | - |  |
|  |  |  |  |  |  |  |  |  |  |
| Eos | OVA-induced | 6 | 45 | 73% | <0.001 | -3.74 | [-5.30,-2.18] | <0.001 | 0.04 |
|  | Df-induced | 1 | 5 | - | - | -10.12 | [-15.95,-4.29] | <0.001 |  |
|  |  |  |  |  |  |  |  |  |  |
| IL-4 | OVA-induced | 5 | 41 | 94% | <0.001 | -5.29 | [-9.21,-1.37] | <0.001 | 0.37 |
|  | DNCB-induced | 1 | 7 | - | - | -3.12 | [-4.84,-1.40] | <0.001 |  |
|  | Df-induced | 1 | 5 | - | - | -5.3 | [-8.53,-2.07] | 0.001 |  |
|  |  |  |  |  |  |  |  |  |  |
| IL-5 | OVA-induced | 4 | 27 | 95% | <0.001 | -5.07 | [-11.86,1.72] | 0.14 | 0.43 |
|  | Df-induced | 1 | 5 | - | - | -8.43 | [-13.33,-3.52] | <0.001 |  |
|  |  |  |  |  |  |  |  |  |  |
| IL-10 | OVA-induced | 3 | 19 | 91% | <0.001 | 1.65 | [-3.38,6.69] | 0.52 | - |
|  | - | 0 | 0 | - | - | - | - | - |  |
|  |  |  |  |  |  |  |  |  |  |
| TNF-α | OVA-induced | 6 | 46 | 93% | <0.001 | -3.17 | [-6.06,-0.29] | 0.03 | - |
|  | - | 0 | 0 | - | - | - | - | - |  |
|  |  |  |  |  |  |  |  |  |  |
| IFN-γ | OVA-induced | 4 | 29 | 90% | <0.001 | 2.75 | [0.17,5.32] | 0.04 | - |
|  | - | 0 | 0 | - | - | - | - | - |  |
|  |  |  |  |  |  |  |  |  |  |
| HIS | OVA-induced | 2 | 20 | 0% | 0.49 | -5.74 | [-7.29,-4.20] | <0.001 | <0.001 |
|  | CPE-induced | 1 | 7 | - | - | -1.72 | [-3.01,-0.43] | <0.001 |  |

n (k) = number of studies; N = total number of animals.
