# Supplementary material for: Quercetin exhibits multi-target anti-allergic effects in animal models: a systematic review and meta-analysis of preclinical studies
Source: Front Pharmacol. 2025 Nov 20;16:1673712. doi: 10.3389/fphar.2025.1673712 (PMC12676024; doi:10.3389/fphar.2025.1673712)
Supplement: Supplementary file 5 [file Table9.docx]

**Table 7.**Subgroup analysis by animal species

| **Outcome** | **Subgroup** | **n(k)** | **N** | **I^2^** | **P(het)** | **SMD** | **95%CI** | **P(effect)** | **P(between)** |
| --- | --- | --- | --- | --- | --- | --- | --- | --- | --- |
| IgE | Mice | 7 | 44 | 94% | <0.001 | -4.67 | [-8.81,-0.53] | 0.03 | 0.11 |
|  | Rats | 1 | 6 | - | - | -1.17 | [-2.44,0.10] | 0.07 |  |
|  |  |  |  |  |  |  |  |  |  |
| OVA-IgE | Mice | 3 | 22 | 75% | 0.02 | -2.97 | [-5.31,-0.62] | 0.01 | 0.48 |
|  | Rats | 2 | 16 | 86% | <0.001 | -4.68 | [-8.84,-0.52] | 0.03 |  |
|  |  |  |  |  |  |  |  |  |  |
| Mac | Mice | 3 | 23 | 0% | 0.58 | -3.25 | [-4.23,-2.28] | <0.001 | <0.001 |
|  | Rats | 1 | 6 | - | - | -0.16 | [-1.30,0.97] | 0.78 |  |
|  |  |  |  |  |  |  |  |  |  |
| Lym | Mice | 3 | 23 | 0% | 0.47 | -4.02 | [-5.15,-2.89] | <0.001 | <0.001 |
|  | Rats | 1 | 6 | - | - | -0.62 | [-1.80,0.55] | 0.3 |  |
|  |  |  |  |  |  |  |  |  |  |
| Neu | Mice | 3 | 23 | 94% | <0.001 | -2.54 | [-6.77,1.70] | 0.24 | 0.39 |
|  | Rats | 1 | 6 | - | - | -0.60 | [-1.77,0.57] | 0.31 |  |
|  |  |  |  |  |  |  |  |  |  |
| Eos | Mice | 6 | 44 | 94% | <0.001 | -4.67 | [-8.81,-0.53] | 0.03 | 0.11 |
|  | Rats | 1 | 6 | - | - | -1.17 | [-2.44,0.10] | 0.07 |  |
|  |  |  |  |  |  |  |  |  |  |
| IL-4 | Mice | 6 | 47 | 93% | <0.001 | -5.01 | [-8.25,-1.77] | <0.001 | 0.63 |
|  | Rats | 1 | 6 | - | - | -4.04 | [-6.32,-1.77] | <0.001 |  |
|  |  |  |  |  |  |  |  |  |  |
| IL-5 | Mice | 5 | 32 | 94% | <0.001 | -5.71 | [-11.82,0.39] | 0.07 | - |
|  | Rats | 0 | 0 | - | - | - | - | - |  |
|  |  |  |  |  |  |  |  |  |  |
| IL-10 | Mice | 2 | 12 | 95% | <0.001 | -0.17 | [-9.88,9.54] | 0.97 | 0.34 |
|  | Rats | 1 | 7 | - | - | 4.74 | [2.42,7.06] | <0.001 |  |
|  |  |  |  |  |  |  |  |  |  |
| TNF-α | Mice | 5 | 39 | 95% | <0.001 | -3.75 | [-7.64,0.14] | 0.06 | 0.31 |
|  | Rats | 1 | 7 | - | - | -1.63 | [-2.90,-0.36] | 0.01 |  |
|  |  |  |  |  |  |  |  |  |  |
| IFN-γ | Mice | 3 | 23 | 92% | <0.001 | 4.35 | [-0.16,8.87] | 0.06 | 0.08 |
|  | Rats | 1 | 6 | - | - | 0.19 | [-0.94,1.33] | 0.74 |  |
|  |  |  |  |  |  |  |  |  |  |
| HIS | Mice | 2 | 20 | 0% | 0.49 | -5.74 | [-7.29,-4.20] | <0.001 | <0.001 |
|  | Rats | 1 | 7 | - | - | -1.72 | [-3.01,-0.43] | <0.001 |  |

n (k) = number of studies; N = total number of animals.
