# Supplementary material for: Quercetin exhibits multi-target anti-allergic effects in animal models: a systematic review and meta-analysis of preclinical studies
Source: Front Pharmacol. 2025 Nov 20;16:1673712. doi: 10.3389/fphar.2025.1673712 (PMC12676024; doi:10.3389/fphar.2025.1673712)
Supplement: Supplementary file 6 [file Table2.docx]

| **Element** | **Description** |
| --- | --- |
| **Population (P)** | Restricted to animal models of allergic diseases.(Food allergy; Allergic rhinitis; Asthma; Allergy-related contact dermatitis; Allergic conjunctivitis; Atopic dermatitis; Allergic airway disease;Anaphylactic shock;) |
| **Intervention (I)** | Quercetin, isolated from Quercus sp. [Fagaceae; Quercus cortex] treatment. |
| **Comparator (C)** | Receiving either no treatment or an equivalent volume of sterile solution. |
| **Outcomes (O)** | Primary: total immunoglobulin E (IgE), ovalbumin-specific IgE (OVA-IgE), cytokine levels (IL-4, IL-5, IL-10,TNF-α(TNF), IFN-γ(IFN)), and immune cell counts (macrophages [Mac], lymphocytes [Lym], eosinophils [Eos], neutrophils [Neu]), and histamine(HIS) |
| **Timing (T)** | Duration of interventions ranged from 14 days to 60 days. |
| **Study Design (S)** | Randomized Controlled Trials (RCTs), double-blind or single-blind, parallel or crossover designs. |
