# Supplementary material for: Quercetin exhibits multi-target anti-allergic effects in animal models: a systematic review and meta-analysis of preclinical studies
Source: Front Pharmacol. 2025 Nov 20;16:1673712. doi: 10.3389/fphar.2025.1673712 (PMC12676024; doi:10.3389/fphar.2025.1673712)
Supplement: Supplementary file 7 [file Table5.docx]

**Table 3.**Subgroup analysis by administration route indicated

| **Outcome** | **Subgroup** | **n(k)** | **N** | **I^2^** | **P(het)** | **SMD** | **95%CI** | **P(effect)** | **P(between)** |
| --- | --- | --- | --- | --- | --- | --- | --- | --- | --- |
| IgE | Intraperitoneal injections | 6 | 52 | 90% | <0.001 | -4.56 | [-6.83,-2.28] | <0.001 | 0.75 |
|  | Others | 2 | 12 | 64% | 0.09 | -3.92 | [-7.10,-0.73] | 0.02 |  |
|  |  |  |  |  |  |  |  |  |  |
| OVA-IgE | Intraperitoneal injections | 4 | 34 | 77% | <0.001 | -3.34 | [-5.03,-1.65] | <0.001 | 0.02 |
|  | Others | 1 | 4 | - | - | -16.79 | [-28.43,-5.16] | <0.001 |  |
|  |  |  |  |  |  |  |  |  |  |
| Mac | Intraperitoneal injections | 4 | 29 | 83% | <0.001 | -2.52 | [-4.43,-0.61] | 0.01 | - |
|  | - | 0 | 0 | - | - | - | - | - |  |
|  |  |  |  |  |  |  |  |  |  |
| Lym | Intraperitoneal injections | 4 | 29 | 83% | <0.001 | -3.24 | [-5.43,-1.04] | <0.001 | - |
|  | - | 0 | 0 | - | - | - | - | - |  |
|  |  |  |  |  |  |  |  |  |  |
| Neu | Intraperitoneal injections | 4 | 29 | 91% | <0.001 | -1.96 | [-4.53,0.60] | 0.13 | - |
|  | - | 0 | 0 | - | - | - | - | - |  |
|  |  |  |  |  |  |  |  |  |  |
| Eos | Intraperitoneal injections | 5 | 41 | 75% | <0.001 | -3.48 | [-5.05,-1.90] | <0.001 | 0.02 |
|  | Others | 2 | 9 | 0% | 0.41 | -8.24 | [-12.01,-4.47] | <0.001 |  |
|  |  |  |  |  |  |  |  |  |  |
| IL-4 | Intraperitoneal injections | 5 | 41 | 94% | <0.001 | -5.29 | [-9.21,-1.37] | <0.001 | 0.49 |
|  | Others | 2 | 12 | 26% | 0.24 | -3.77 | [-5.71,-1.82] | <0.001 |  |
|  |  |  |  |  |  |  |  |  |  |
| IL-5 | Intraperitoneal injections | 3 | 23 | 96% | <0.001 | -5.46 | [-15.34,4.42] | 0.28 | 0.93 |
|  | Others | 2 | 9 | 48% | 0.16 | -5.92 | [-9.92,-1.92] | <0.001 |  |
|  |  |  |  |  |  |  |  |  |  |
| IL-10 | Intraperitoneal injections | 2 | 15 | 0% | 0.96 | 4.69 | [3.13,6.24] | <0.001 | <0.001 |
|  | Others | 1 | 4 | - | - | -5.27 | [-9.15,-1.39] | <0.001 |  |
|  |  |  |  |  |  |  |  |  |  |
| TNF-α | Intraperitoneal injections | 5 | 42 | 94% | <0.001 | -2.46 | [-5.38,0.45] | 0.1 | 0.04 |
|  | Others | 1 | 4 | - | - | -11.17 | [-18.97,-3.36] | <0.001 |  |
|  |  |  |  |  |  |  |  |  |  |
| IFN-γ | Intraperitoneal injections | 4 | 29 | 90% | <0.001 | 2.75 | [0.17,5.32] | 0.04 | - |
|  | - | 0 | 0 | - | - | - | - | - |  |
|  |  |  |  |  |  |  |  |  |  |
| HIS | Intraperitoneal injections | 3 | 27 | 87% | <0.001 | -4.4 | [-7.48,-1.31] | <0.001 | - |
|  | - | 0 | 0 | - | - | - | - | - |  |

n (k) = number of studies; N = total number of animals.
