# Supplementary material for: Quercetin exhibits multi-target anti-allergic effects in animal models: a systematic review and meta-analysis of preclinical studies
Source: Front Pharmacol. 2025 Nov 20;16:1673712. doi: 10.3389/fphar.2025.1673712 (PMC12676024; doi:10.3389/fphar.2025.1673712)
Supplement: Supplementary file 9 [file Table3.docx]

**Table 2.** Characteristics of the studies included in the meta-analysis.

| **Author** | **Country** | **Year** | **Disease** | **Formulation** | **Animal** | **Sex** | **Per-Arm Sample Sizes** | **Age/Weight** | **Intervention** | **Route** | **Dose** | **Frequency** | **Duration** | **Outcome** | **Matrix** | **Randomization** | **Blinding** |
| --- | --- | --- | --- | --- | --- | --- | --- | --- | --- | --- | --- | --- | --- | --- | --- | --- | --- |
| E. Zhou | China | 2024 | Food allergy | Covalent conjugate | Mice | Female | 8 | A:6 weeks old  W:16 - 20 g | C: OVA solution  T: OVA solution + PQ-treated | intraperitoneal injections | 1.11 mg/kg | 1 times a week; 4 weeks | 28 days | IgE; IL-4; IL-10;  HIS | IgE; HIS: serum  IL-4; IL-10: tissue | Yes | Unclear |
| 1. Mu | China | 2024 | Allergic rhinitis | Aglycone | Mice | Male | 5 | A:6 weeks old  W:18 - 20 g | C: OVA solution  T: OVA solution + QU-treated | intraperitoneal injections | Unclear | Freq:4 times; days 1. 5. 10. 14 | 42 days | IgE; TNF-α | IgE: serum  TNF-α: BALF | Yes | Yes |
| M.A. Rajizadeh | Iran | 2023 | Asthma | Aglycone | Wistar rats | Male | 7 | A:8 weeks old  W:200-250 g | C: OVA solution  T: OVA solution + QS-treated | intraperitoneal injections | 50 mg/kg | Freq: once a day; 7 days | 50 days | IL-10; TNF-α | tissue | Yes | Yes |
| L.W.Chen | Taiwan | 2021 | Asthma | Glycoside | Mice | Female | 10 | A:8-12 weeks old | C: OVA solution  T: OVA solution + QU-treated | intraperitoneal injections | 100 μmol/kg | Freq: once a day; 32 days | 33 days | IgE; ova-IgE;  IL-4; IL-5; Mac;Lym;Eos;Neu;TNF-α; IFN-γ | IgE; ova-IgE: serum  IL-4; IL-5; TNF-α; IFN-γ;Mac;Lym; Eos; Neu: BALF | Yes | Yes |
| J. Jegal | Korea | 2020 | Allergy-related contact dermatitis | Glycoside | SKH-1 hairless mice | Female | 7 | A:6 weeks old | C: DNCB plus vehicle  T: DNCB sensitized plus treatment with quercitrin | dorsal skins | 40–50 mg/kg | Freq: alternate days; 2 weeks | 22 days | IgE; IL-4 | serum | Yes | Unclear |
| Y. Ding | China | 2019 | Allergic conjunctivitis | Aglycone | C57BL/6 mice | Male | 12 | W:25-30 g | C: OVA solution  T: OVA solution + QU-treated | intraperitoneal injections | 4 mg/kg | Freq: once a day; 3 weeks | 38 days | IgE; IL-4; Eos; TNF-α; HIS | IgE; IL-4; TNF-α: serum  Eos; HIS: tissue | Yes | Unclear |
| M. Sagit | Turkey | 2017 | Allergic rhinitis | Aglycone | SD rats | Female | 10 | A:20-22 weeks old  W:200-250 g | C: OVA solution  T: OVA solution + QU-treated | intraperitoneal injections | 80 mg/kg | Freq: alternate days; 2 weeks | 21 days | IgE; ova-IgE | serum | Yes | Yes |
| Y. Cai | China | 2017 | Asthma | Aglycone | SD rats | Unclear | 6 | W:180-220 g | C: OVA solution  T: OVA solution + QU-treated | intraperitoneal injections | 50 mg/kg | Freq: once a day; 14 days | 14 days | ova-IgE; IL-4;  Mac; Lym; Eos; Neu; IFN-γ | ova-IgE: serum  IL-4; Mac; Lym; Eos; Neu; IFN-γ:BALF | Yes | Unclear |
| E.J.Park | Korea | 2014 | Atopic dermatitis | Glycoside | NC/Nga mice | Female | 5 | A:3 weeks old | C: Df ointment  T: Df ointment + QGR-treated | dorsal skins | Unclear | Freq: twice a week; for 4 weeks | 56 days | IgE; IL-4; IL-5; Eos | serum | Yes | Unclear |
| E.A. Cruz | Germany | 2012 | Allergic airway disease | Glycoside | BALB/c mice | Unclear | 8 | A:8-12 weeks old | C: OVA solution  T: OVA solution + QI-treated | intraperitoneal injections | 30 mg/kg | Freq: 2 times; days 0.14 | 30 days | ova-IgE; IL-5;  Mac; Lym; Eos; Neu;  TNF-α; IFN-γ | ova-IgE; IL-5;TNF-α; IFN-γ: serum  Mac; Lym; Eos; Neu: BALF | Yes | Yes |
| F.Shishehbor | Iran | 2010 | Food allergy | Aglycone | Wistar rats | Male | 7 | A:4-6 weeks old  W:70 - 130 g | C: CPE solution  T: CPE solution + QU-treated | intraperitoneal injections | 50 mg/kg | Freq: 6 times; days 8.9.16.17.24.25 | 60 days | IgE; HIS | serum | Yes | Unclear |
| H.J. Park | Korea | 2009 | Asthma | Aglycone | BALB/c mice | Male | 5 | A:6-8 weeks old | C: OVA solution  T: OVA solution + QU-treated | intraperitoneal injections | 16 mg/kg | Freq: 2 times; days 1.15 | 24 days | IL-4; IL-5;  Mac; Lym; Eos; Neu;  IFN-γ | IL-4; IL-5: BALF  Mac; Lym; Neu; IFN-γ: serum  Eos: tissue | Yes | Yes |
| E.A. Cruz | Brazil | 2008 | Anaphylactic shock | Glycoside | BALB/c mice | Male | 8 | W:18 - 20 g | C: OVA solution  T: OVA solution + QU-treated | injections in the rump | 20 mg/kg | Freq: 1 times a week; 3weeks | 14 days | ova-IgE; IL-5; IL-10; Eos; TNF-α | ova-IgE; Eos:serum  IL-5; IL-10;  TNF-α: tissue | Yes | Unclear |

T: experimental group; C: control group; HIS: Histamine; Mac: macrophages; Lym: lymphocytes; Neu,:neutrophils; Eos: eosinophils; IL: interleukin;

TNF-α: tumor necrosis factor-α; IFN- γ: interferoneinterferone-γ

PQ: Bra c p-quercetin covalent polymer; QU: Quercetin; QS: Quercetin; QI: Quercitrin,

DNCB: Dinitrochlorobenzene; DF: Dermatophagoides farina; CPE: crude PN extract; QGR:Quercetin-3-O-(200-gallate)-a-l-rhamnopyranoside
