# Supplementary material for: Quercetin exhibits multi-target anti-allergic effects in animal models: a systematic review and meta-analysis of preclinical studies
Source: Front Pharmacol. 2025 Nov 20;16:1673712. doi: 10.3389/fphar.2025.1673712 (PMC12676024; doi:10.3389/fphar.2025.1673712)
Supplement: Supplementary file 10 [file Table4.docx]

Table 14

| **Bias Domain** | **Yes (Low Risk)** | **Unclear (Some Concerns)** | **No (High Risk)** |
| --- | --- | --- | --- |
| Sequence Generation | 1 (7.7%) | 12 (92.3%) | 0 (0%) |
| Baseline Characteristics | 13 (100%) | 0 (0%) | 0 (0%) |
| Allocation Concealment | 0 (0%) | 13 (100%) | 0 (0%) |
| Random Housing | 0 (0%) | 13 (100%) | 0 (0%) |
| Blinding of Personnel | 0 (0%) | 0 (0%) | 13 (100%) |
| Random Outcome Assessment | 1 (7.7%) | 12 (92.3%) | 0 (0%) |
| Blinding of Outcome Assessors | 8 (61.5%) | 0 (0%) | 5 (38.5%) |
| Incomplete Data Reporting | 13 (100%) | 0 (0%) | 0 (0%) |
| Selective Outcome Reporting | 13 (100%) | 0 (0%) | 0 (0%) |
| Other Biases | 1 (7.7%) | 12 (92.3%) | 0 (0%) |

Note: n=13 studies.
