# Supplementary material for: Quercetin exhibits multi-target anti-allergic effects in animal models: a systematic review and meta-analysis of preclinical studies
Source: Front Pharmacol. 2025 Nov 20;16:1673712. doi: 10.3389/fphar.2025.1673712 (PMC12676024; doi:10.3389/fphar.2025.1673712)
Supplement: Supplementary file 11 [file Table6.docx]

**Table 4.**Subgroup analysis by treatment duration

| **Outcome** | **Subgroup** | **n(k)** | **N** | **I^2^** | **P(het)** | **SMD** | **95%CI** | **P(effect)** | **P(between)** |
| --- | --- | --- | --- | --- | --- | --- | --- | --- | --- |
| IgE | <30 days | 3 | 25 | 93% | <0.001 | -9.76 | [-18.41,-1.11] | 0.03 | 0.11 |
|  | >=30 days | 5 | 39 | 74% | <0.001 | -2.57 | [-3.92,-1.21] | <0.001 |  |
|  |  |  |  |  |  |  |  |  |  |
| OVA-IgE | <30 days | 3 | 20 | 83% | <0.001 | -6.17 | [-10.82,-1.52] | <0.001 | 0.13 |
|  | >=30 days | 2 | 18 | 50% | 0.16 | -2.39 | [-3.68,-1.10] | <0.001 |  |
|  |  |  |  |  |  |  |  |  |  |
| Mac | <30 days | 2 | 11 | 86% | <0.001 | -1.83 | [-5.41,1.76] | 0.32 | 0.49 |
|  | >=30 days | 2 | 18 | 0% | 0.36 | -3.15 | [-4.21,-2.09] | <0.001 |  |
|  |  |  |  |  |  |  |  |  |  |
| Lym | <30 days | 2 | 11 | 84% | 0.01 | -2.42 | [-6.37,1.52] | 0.23 | 0.47 |
|  | >=30 days | 2 | 18 | 21% | 0.26 | -3.96 | [-5.38,-2.53] | <0.001 |  |
|  |  |  |  |  |  |  |  |  |  |
| Neu | <30 days | 2 | 11 | 76% | 0.04 | -1.7 | [-4.17,0.77] | 0.18 | 0.87 |
|  | >=30 days | 2 | 18 | 97% | <0.001 | -2.29 | [-8.77,4.20] | 0.49 |  |
|  |  |  |  |  |  |  |  |  |  |
| Eos | <30 days | 3 | 15 | 83% | <0.001 | -4.84 | [-9.76,0.08] | 0.05 | 0.79 |
|  | >=30 days | 4 | 35 | 57% | 0.07 | -4.15 | [-5.69,-2.60] | <0.001 |  |
|  |  |  |  |  |  |  |  |  |  |
| IL-4 | <30 days | 4 | 26 | 0% | 0.79 | -3.8 | [-4.84,-2.76] | <0.001 | 0.45 |
|  | >=30 days | 3 | 27 | 96% | <0.001 | -7.15 | [-15.76,1.46] | 0.1 |  |
|  |  |  |  |  |  |  |  |  |  |
| IL-5 | <30 days | 2 | 9 | 53% | 0.14 | -6.07 | [-10.40,-1.74] | <0.001 | 0.89 |
|  | >=30 days | 3 | 23 | 96% | <0.001 | -5.35 | [-15.08,4.38] | 0.28 |  |
|  |  |  |  |  |  |  |  |  |  |
| IL-10 | <30 days | 2 | 12 | 95% | <0.001 | -0.17 | [-9.88,9.54] | 0.97 | 0.34 |
|  | >=30 days | 1 | 7 | - | - | 4.74 | [2.42,7.06] | <0.001 |  |
|  |  |  |  |  |  |  |  |  |  |
| TNF-α | <30 days | 1 | 4 | - | - | -11.17 | [-18.97,-3.36] | <0.001 | 0.04 |
|  | >=30 days | 5 | 42 | 94% | <0.001 | -2.46 | [-5.38,0.45] | 0.1 |  |
|  |  |  |  |  |  |  |  |  |  |
| IFN-γ | <30 days | 2 | 11 | 91% | 0.001 | 4.51 | [-4.71,13.73] | 0.34 | 0.72 |
|  | >=30 days | 2 | 18 | 94% | <0.001 | 2.61 | [-2.02,7.23] | 0.27 |  |
|  |  |  |  |  |  |  |  |  |  |
| HIS | <30 days | 1 | 8 | - | - | -6.58 | [-9.38,-3.78] | <0.001 | 0.18 |
|  | >=30 days | 2 | 19 | 90% | 0.001 | -3.49 | [-7.08,0.10] | 0.06 |  |

n (k) = number of studies; N = total number of animals.
