# Supplementary material for: Quercetin exhibits multi-target anti-allergic effects in animal models: a systematic review and meta-analysis of preclinical studies
Source: Front Pharmacol. 2025 Nov 20;16:1673712. doi: 10.3389/fphar.2025.1673712 (PMC12676024; doi:10.3389/fphar.2025.1673712)
Supplement: Supplementary file 12 [file Table10.docx]

**Table 8.**Subgroup analysis by disease model

| **Outcome** | **Subgroup** | **n(k)** | **N** | **I^2^** | **P(het)** | **SMD** | **95%CI** | **P(effect)** | **P(between)** |
| --- | --- | --- | --- | --- | --- | --- | --- | --- | --- |
| IgE | Food allergy | 2 | 15 | 95% | <0.001 | -7.67 | [-20.50,5.17] | 0.24 | 0.84 |
|  | Allergic rhinitis | 2 | 15 | 96% | <0.001 | -6.7 | [-18.58,5.18] | 0.27 |  |
|  | Atopic dermatitis | 2 | 12 | 64% | 0.09 | -3.92 | [-7.10,-0.73] | 0.02 |  |
|  | Others | 2 | 22 | 57% | 0.13 | -3.26 | [-4.79,-1.73] | <0.001 |  |
|  |  |  |  |  |  |  |  |  |  |
| OVA-IgE | Asthma | 2 | 16 | 0% | 0.7 | -2.93 | [-4.01,-1.85] | <0.001 | 0.02 |
|  | Allergic rhinitis | 1 | 10 | - | - | -6.92 | [-9.46,-4.37] | <0.001 |  |
|  | Others | 2 | 12 | 84% | 0.01 | -8.12 | [-22.67,6.42] | 0.27 |  |
|  |  |  |  |  |  |  |  |  |  |
| Mac | Asthma | 3 | 21 | 84% | <0.001 | -2.1 | [-4.30,0.11] | 0.06 | 0.23 |
|  | Others | 1 | 8 | - | - | -3.84 | [-5.65,-2.02] | <0.001 |  |
|  |  |  |  |  |  |  |  |  |  |
| Lym | Asthma | 3 | 21 | 84% | <0.001 | -2.69 | [-5.10,-0.28] | 0.03 | 0.17 |
|  | Others | 1 | 8 | - | - | -4.95 | [-7.16,-2.75] | <0.001 |  |
|  |  |  |  |  |  |  |  |  |  |
| Neu | Asthma | 3 | 21 | 89% | <0.001 | -3.04 | [-6.14,0.06] | 0.05 | 0.02 |
|  | Others | 1 | 8 | - | - | 0.95 | [-0.10,2.01] | 0.08 |  |
|  |  |  |  |  |  |  |  |  |  |
| Eos | Asthma | 3 | 21 | 85% | 0.001 | -3.78 | [-6.85,-0.72] | 0.02 | 0.6 |
|  | Others | 4 | 29 | 64% | 0.04 | -4.8 | [-7.06,-2.54] | <0.001 |  |
|  |  |  |  |  |  |  |  |  |  |
| IL-4 | Asthma | 2 | 12 | 26% | 0.24 | -3.77 | [-5.71,-1.82] | <0.001 | 0.49 |
|  | Atopic dermatitis | 3 | 21 | 91% | <0.001 | -2.52 | [-6.33,1.29] | 0.19 |  |
|  | Others | 2 | 20 | 95% | <0.001 | -10.83 | [-24.50,2.84] | 0.12 |  |
|  |  |  |  |  |  |  |  |  |  |
| IL-5 | Asthma | 2 | 15 | 94% | <0.001 | -2.83 | [-13.91,8.25] | 0.62 | 0.43 |
|  | Others | 3 | 17 | 66% | 0.05 | -7.56 | [-11.65,-3.47] | <0.001 |  |
|  |  |  |  |  |  |  |  |  |  |
| IL-10 | Asthma | 1 | 7 | - | - | 4.74 | [2.42,7.06] | <0.001 | <0.001 |
|  | Food allergy | 1 | 8 | - | - | 4.65 | [2.55,6.74] | <0.001 |  |
|  | Others | 1 | 4 | - | - | -5.27 | [-9.15,-1.39] | <0.001 |  |
|  |  |  |  |  |  |  |  |  |  |
| TNF-α | Asthma | 2 | 17 | 95% | <0.001 | 0.32 | [-3.49,4.12] | 0.87 | 0.04 |
|  | Others | 4 | 29 | 88% | <0.001 | -5.33 | [-9.02,-1.64] | <0.001 |  |
|  |  |  |  |  |  |  |  |  |  |
| IFN-γ | Asthma | 3 | 21 | 92% | <0.001 | 4.33 | [-0.27,8.93] | 0.07 | 0.1 |
|  | Others | 1 | 8 | - | - | 0.32 | [-0.66,1.31] | 0.52 |  |
|  |  |  |  |  |  |  |  |  |  |
| HIS | Food allergy | 2 | 15 | 90% | <0.001 | -3.98 | [-8.73,0.77] | 0.1 | 0.59 |
|  | Others | 1 | 12 | - | - | -5.38 | [-7.23,-3.53] | <0.001 |  |

n (k) = number of studies; N = total number of animals.
